# Supplementary material for: A pan-tissue DNA-methylation epigenetic clock based on deep learning
Source: NPJ Aging. 2022 Apr 19;8(1):4. doi: 10.1038/s41514-022-00085-y (PMC9158789; doi:10.1038/s41514-022-00085-y)
Supplement: Supplementary file 1 — New SI requested by ME [file 41514_2022_85_MOESM1_ESM.pdf]

Supplementary Information

DeepPINK Results

To support the results obtained by SHAP, we also applied another method of determining feature importance called DeepPINK [**?**]. It works by comparing the original features with fake features. The knockoff features can be generated in many different ways, as long as they simulate the original data structure but are not related to the output. DeepPINK contrasts the relevance of the fake features against the regular input features to determine which ones are truly related to the output. It can also be used for feature selection with a controllable false discovery rate (FDR). It is worth highlighting the difficulty in feature selection in DNA methylation data. Most experiments have a couple dozen or a couple hundred samples. Depending on the type of platform used, the number of beta values for the CpG sites analyzed can vary from around 27 thousand to around 850 thousand. DeepPINK, even with a high FDR of 0.5, only selected 78 features. The fact that other sets of CpG sites unrelated to Horvath's 353 also perform similarly well emphasizes the difficulty in finding the "true" age-related CpG sites.

AltumAge captures relevant age-related CpG-CpG interactions The top 9 most important CpG sites according to SHAP account for 9.54% of total DeepPINK model importance.

Characterization of CpG sites by model interpretation. For the CTCF binding site analysis, the top 1000 CpG sites comprises 47.3% of DeepPINK importance. For the ChromHMM analysis, CpG importance values are also impacted by ChromHMM state for DeepPINK (Kruskal-Wallis H-test p-value = 2.982e-05). The chromatin state with the highest DeepPINK normalized median importance was heterochromatin (DeepPINK importance = 2.47e-14%, top 66th percentile of all CpG sites). Despite there being only 29 CpG sites characterized as heterochromatic, this result emphasizes the importance of chromatin packing with aging, as it is related to genome stability and maintenance.

Aging-related pathways. None of the CpG sites in SIRT genes appear very relevant for DeepPINK. cg21770145, located in SIRT7, accounts for 7.89e-12% of total DeepPINK importance and ranks 1342, with the highest SIRT DeepPINK importance value. For the mTOR pathway, cg05546044, located in MAPK1, has the highest DeepPINK importance of 0.029%, ranking 233. mTOR was not particularly relevant, with its most important CpG site being cg07029998 (DeepPINK importance = 1.12e-12%, rank 2459). All AMPK-related CpG sites had low (less than 10e-13%) DeepPINK importance values.

Figures and Tables

Supplementary Table 1: Evaluation metrics for all models in the validation set. The median absolute error (MAE) and the median error are in units of year, while the mean squared error (MSE) is in units of year-squared. R stands for Pearson's correlation coefficient. Bold numbers indicate better performance.

Model	CpGs	MAE	MSE	R	Median Error
AltumAge	20318	**3.337**	**59.712**	**0.953**	-0.922
AltumAge with ElasticNet CpGs	699	3.516	61.202	0.952	0.207
TabNet	20318	4.027	62.113	0.952	0.003
ElasticNet	699	3.708	67.981	0.947	-0.129
Random Forest	20318	6.206	131.126	0.895	**-0.001**
Support Vector Regression	20318	10.903	280.841	0.780	-2.668

Supplementary Table 2: List of ChromHMM states by ChromHMM state ID.

ChromHMM state ID	ChromHMM state
1	Active TSS
2	Flanking TSS
3	Flanking TSS Upstream
4	Flanking TSS Downstream
5	Strong transcription
6	Weak transcription
7	Genic enhancer1
8	Genic enhancer2
9	Active Enhancer 1
10	Active Enhancer 2
11	Weak Enhancer
12	ZNF genes and repeats
13	Heterochromatin
14	Bivalent/Poised TSS
15	Bivalent Enhancer
16	Repressed PolyComb
17	Weak Repressed PolyComb
18	Quiescent/Low

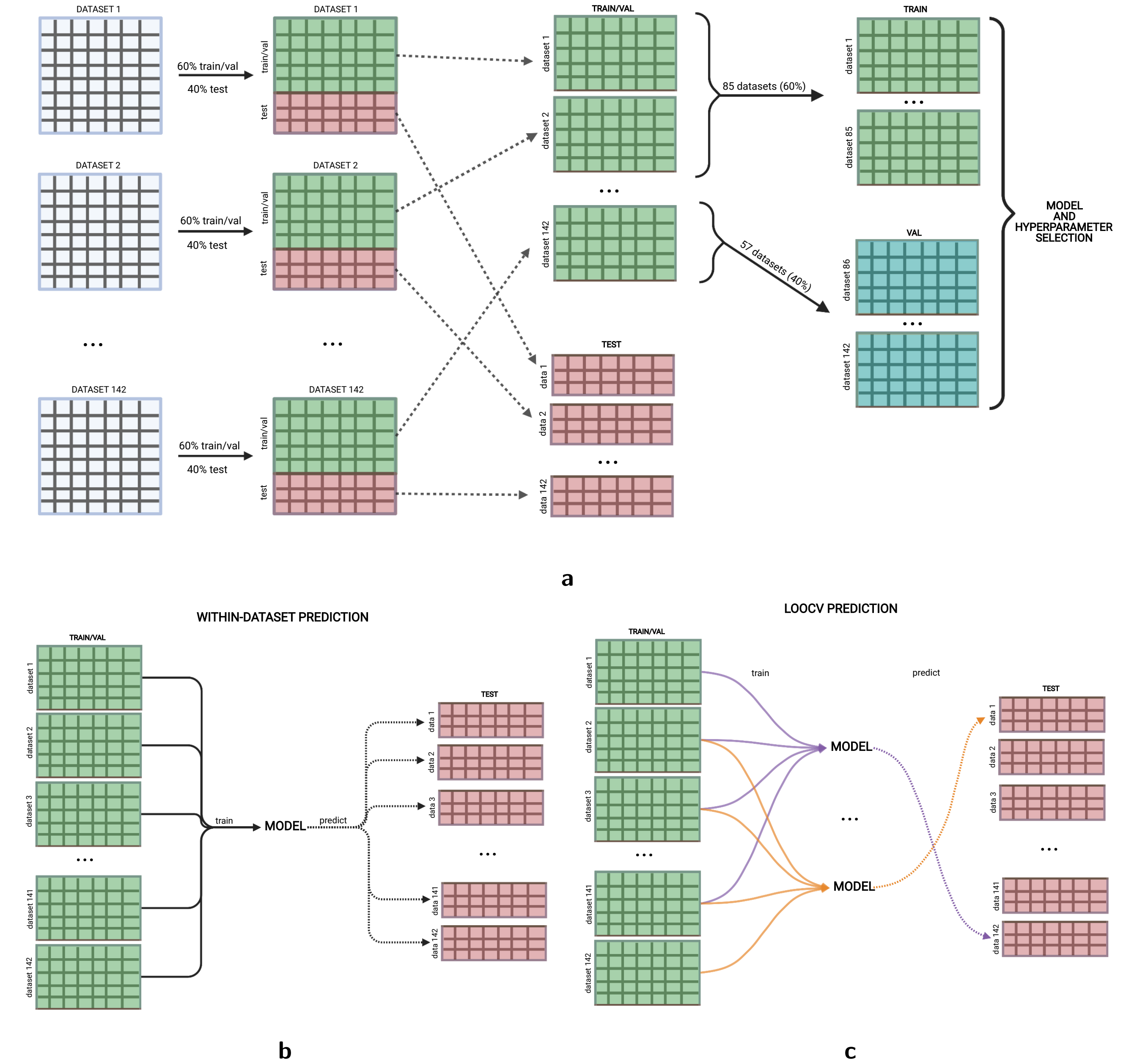

Supplementary Figure 1: Data split workflow. (a) Initially, each data set was randomly split 60% for training and validation and 40% for test. Then, the training data from 85 data sets (60% of training and validation) were used for training and the remaining 57 data sets (40% of training and validation) were used for validation. (b) For within-data set prediction, all training samples were used to train and predicted the entire test set. (c) For leave-one-data set-out cross validation (LOOCV) prediction, the training data from all data sets but one was used to train the model and predict the test set of the left-out data set. This process was repeated 142 times.

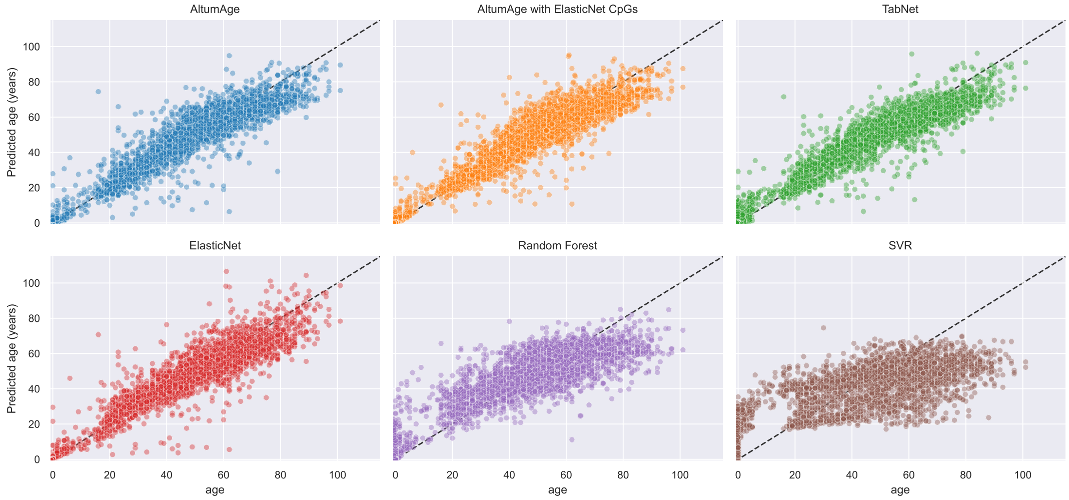

a

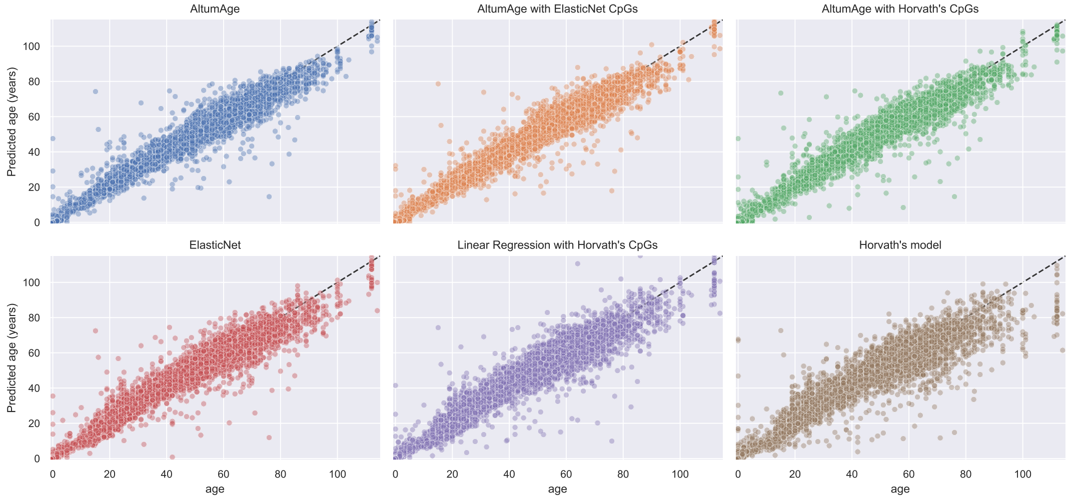

b

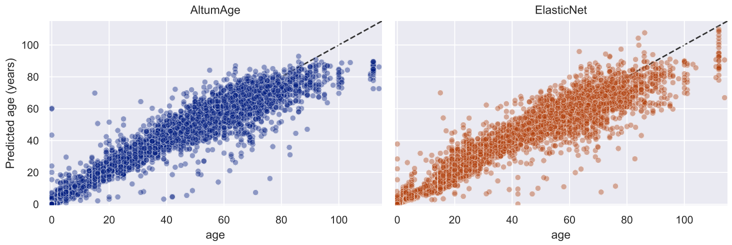

c

Supplementary Figure 2: Qualitative comparison of different models. (a) Models from model selection predicting the validation set. (b) Models from within-data set prediction in the test set. (c) Models from leave-one-data set-out cross validation (LOOCV) in the test set. In each panel, models are ordered from smallest to largest mean squared error (MSE). As shown by the closeness of the predictions to the true age line, AltumAge outperforms other models.

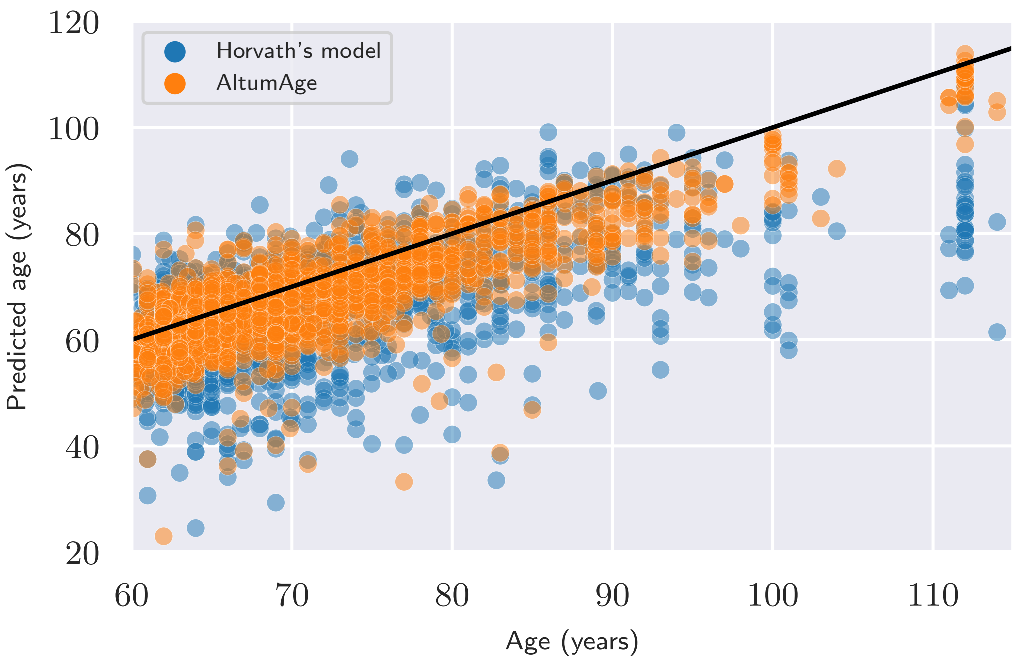

Supplementary Figure 3: Scatter plot showing the improved performance of AltumAge in comparison to Horvath's 2013 model for older ages. The black line represents the location where the predicted age equals the real age. AltumAge's predictions are generally closer to the black line. Horvath's predictions tends to give lower performance in higher ages.

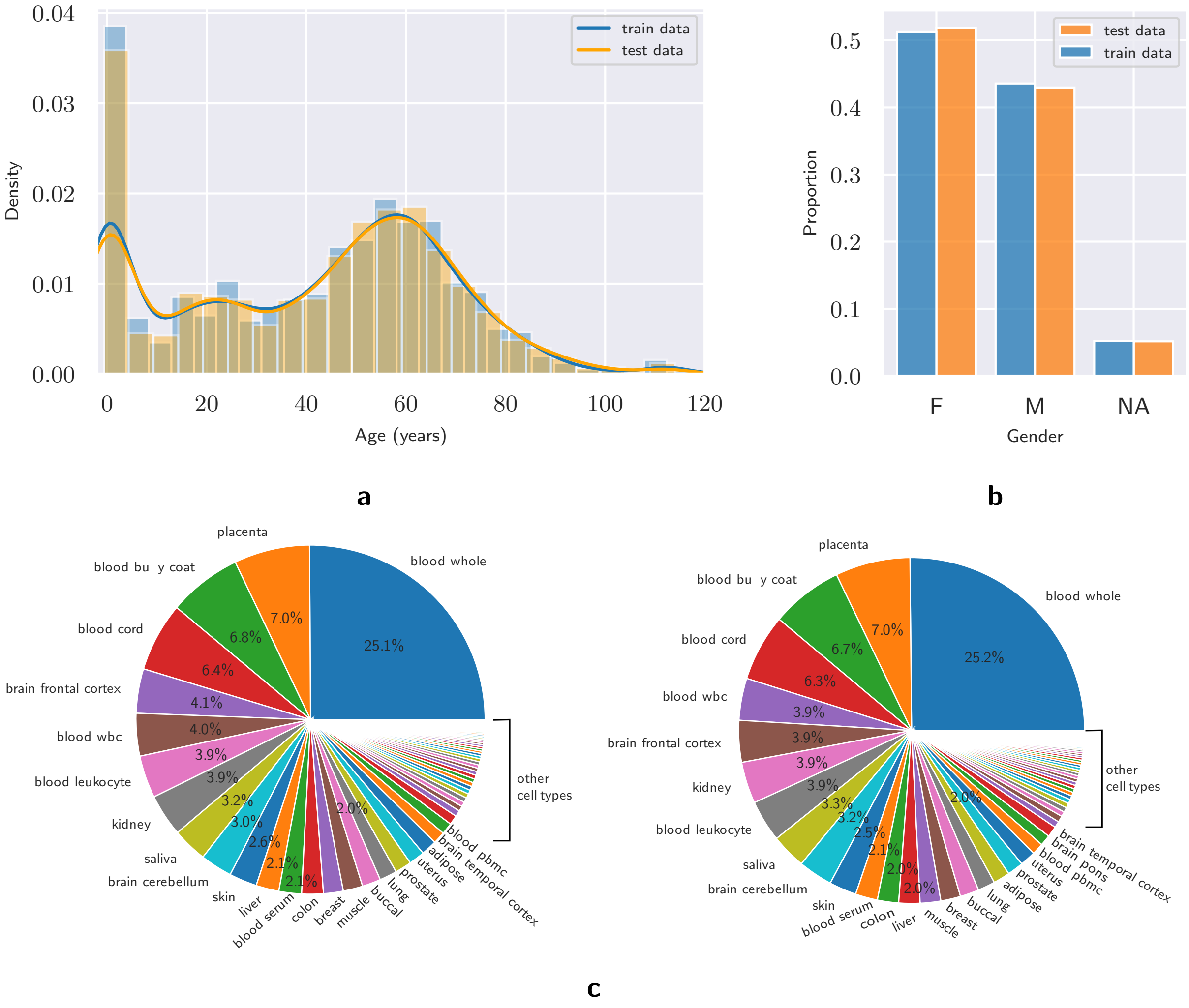

Supplementary Figure 4: Distribution of age, gender, and tissue type in both training and testing sets. The distributions of age (a) and gender (b) are virtually identical between the two sets. Similarly with the distribution of tissue type (c) in training (left) and testing (right).

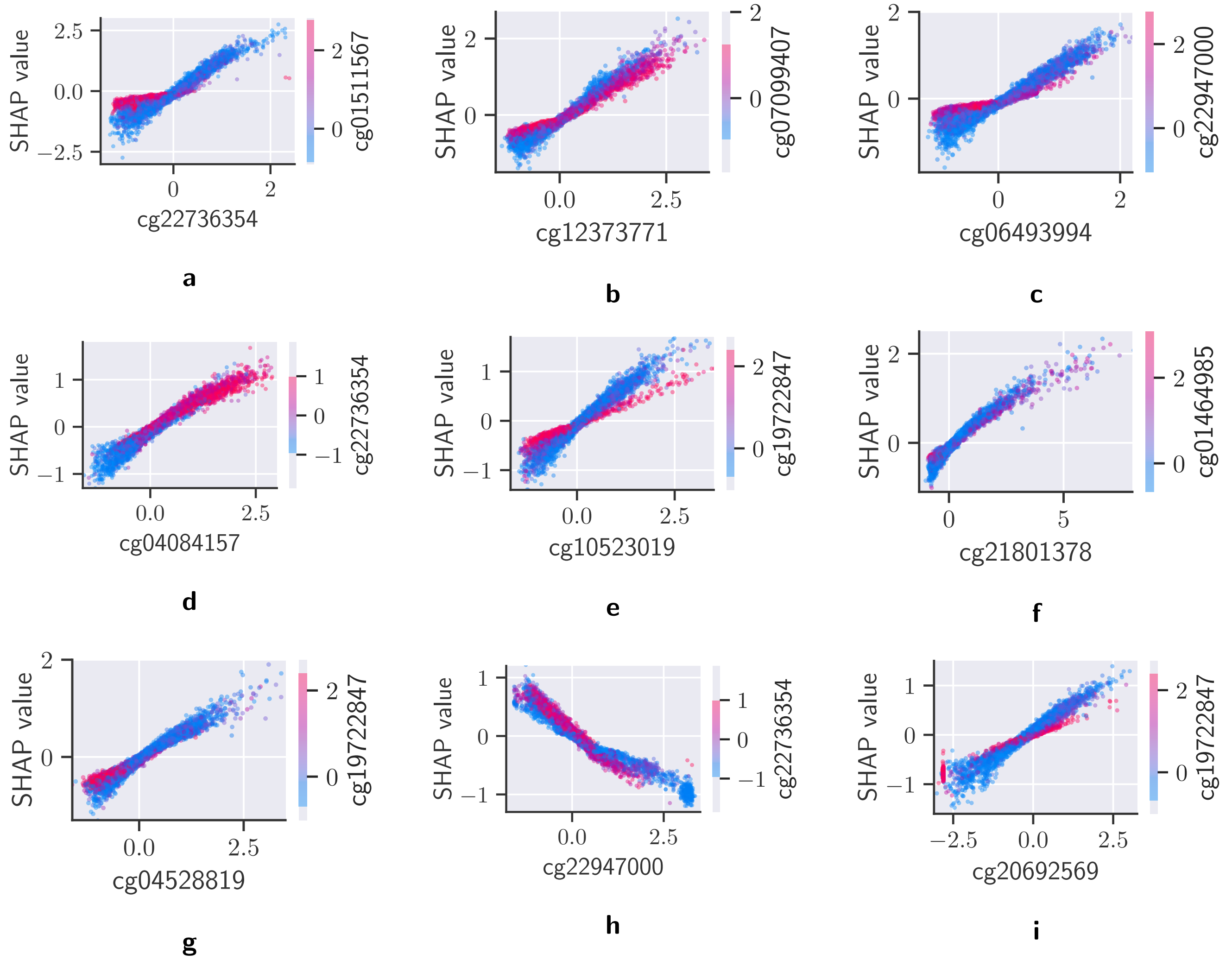

Supplementary Figure 5: Dependence plots of the nine most important CpG sites in AltumAge based on SHAP values. They are ordered from top left to bottom right (a-i) in terms of importance. The x-axis shows the scaled beta values for each specific CpG site; the y-axis, its SHAP value, and the coloring scheme, the scaled beta values for the CpG site with the highest interaction. The effect of a specific CpG site on the predicted age can vary based on a secondary CpG site.

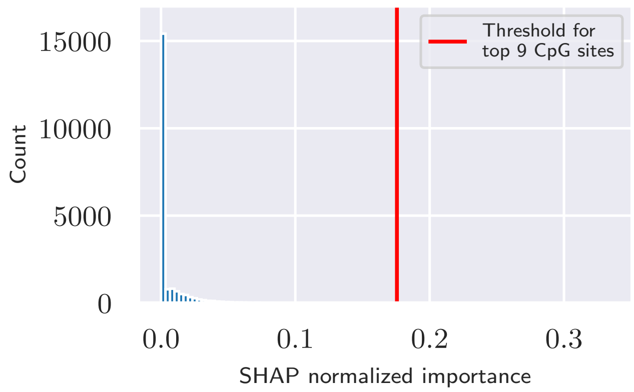

Supplementary Figure 6: Histogram of the normalized importance values of all AltumAge CpG sites according to SHAP. The red line represents the threshold for the top nine CpG sites. These have a much higher importance than most other CpG sites.

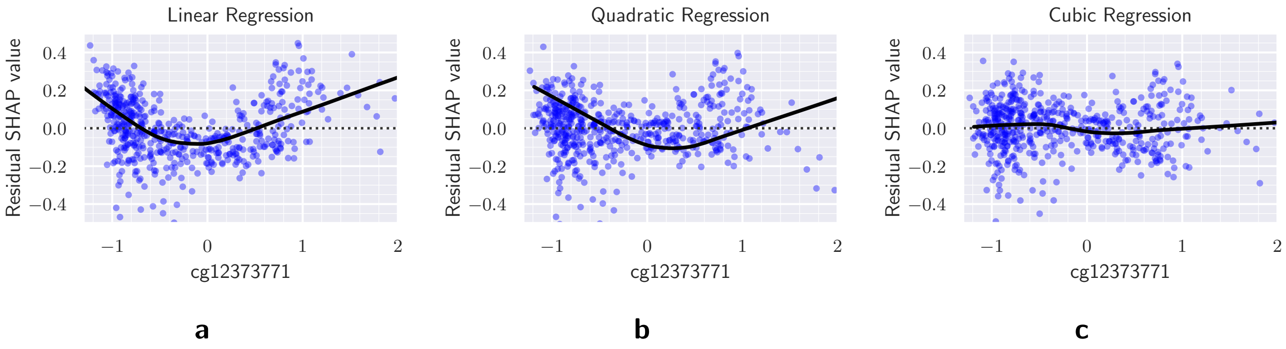

Supplementary Figure 7: Residual plots of cg12373771 by order of regression with lowess line. A linear regression (a) underestimates around the boundaries and underestimates in the middle, demonstrating the non-linear relationship. The same occurs with a quadratic regression (b). When the order is increased to three, the cubic regression (c) takes the non-linearity better into account.

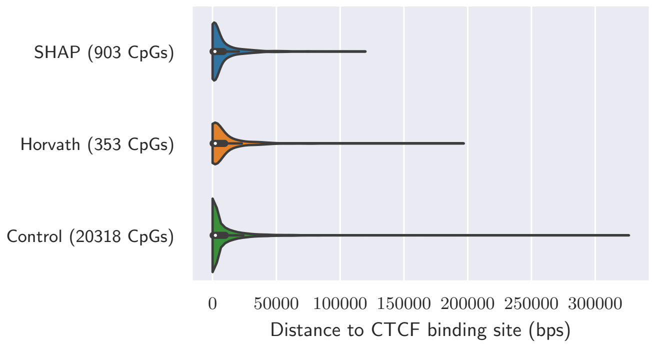

Supplementary Figure 8: Violin plot showing distance of CTCF binding sites for AltumAge's top CpGs based on SHAP and Horvath's CpGs. The top CpG sites according to SHAP are closer to CTCF binding sites than the 20,318 control CpG sites. Horvath's CpG sites are not statistically significantly different from the control.

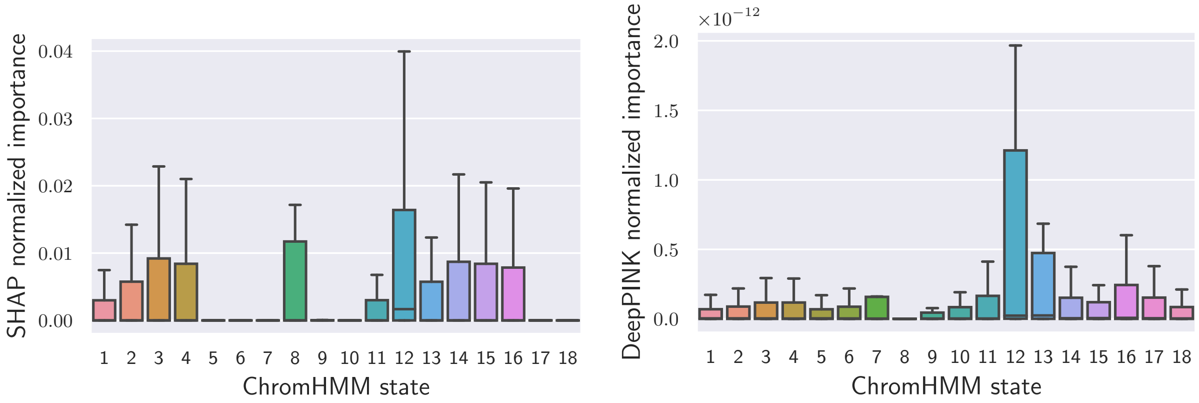

Supplementary Figure 9: Box plots of SHAP and DeepPINK normalized importance values by ChromHMM state. Outliers were removed for better figure visualization. No specific ChromHMM state stands out in importance. The box and whiskers show the quartiles of the data.

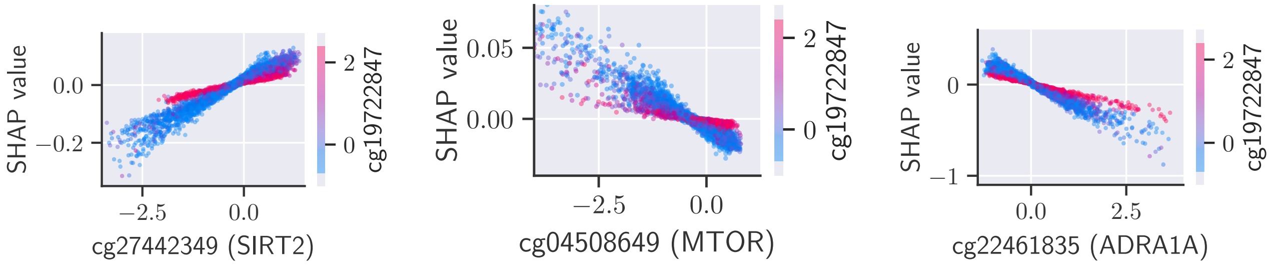

a **b** **c**

Supplementary Figure 10: SHAP dependence plots of three CpG sites in SIRT2, mTOR, and ADRA1A. The x-axis shows the standardized beta values for each specific CpG site; the y-axis, its SHAP value, and the coloring scheme, the scaled beta values for the CpG site with the highest interaction. These are the most important CpG sites according to SHAP for AltumAge in the SIRT and mTOR pathways.

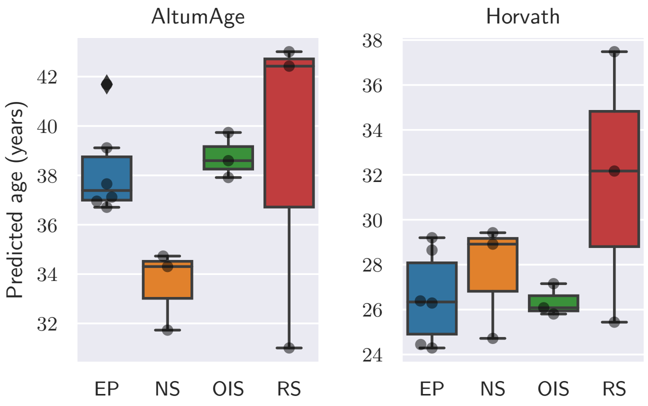

Supplementary Figure 11: Analysis of the effect of cellular senescence in data set GSE91069. Box plots showing predicted age for cultured fibroblasts in early passage (EP), near senescence (NS), oncogene-induced senescence (OIS), and replicative senescence (RS). The box and whiskers show the quartiles of the data.

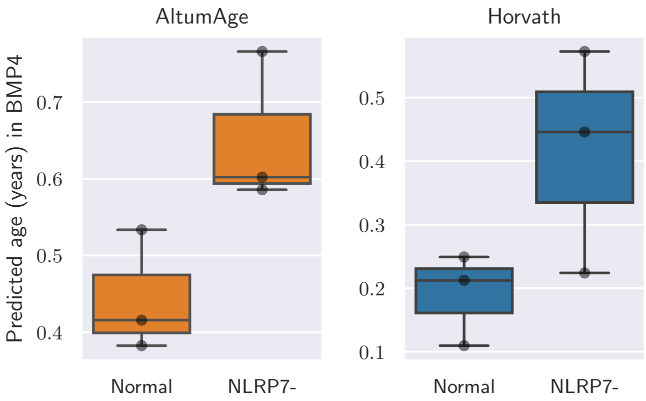

Supplementary Figure 12: Box plots showing predicted age of H9 ESCs with NLRP7 knockdown (NLRP7-) or control (Normal) in BMP4 differentiating medium. The box and whiskers show the quartiles of the data.

SupplementaryTable3

dataset	Tissue type	Data	n	Male proportion	Median age	Std age	MAE (Horvath)	MSE (Horvath)	R (Horvath)	Median error (Horvath)	MAE (AltumAge)	MSE (AltumAge)	R (AltumAge)	Median error (AltumAge)
E-GEOD-21232	pancreas	test	4	1.0	69.5	7.661	13.08	208.647	0.878	13.08	12.876	170.138	0.985	12.876
E-GEOD-27044	blood wbc	test	180	1.0	10.583	4.716	1.269	6.723	0.878	0.054	1.058	3.7	0.914	0.095
E-GEOD-30758	uterus	test	61	0.0	25.0	9.057	6.196	53.033	0.798	-5.388	3.583	23.277	0.872	-0.707
E-GEOD-30759	uterus	test	6	0.0	39.964	7.42	1.036	11.195	0.935	-0.913	6.242	42.681	0.743	-6.242
E-GEOD-30870	blood cord; blood whole	test	16	0.0	90.0	36.617	14.428	172.314	0.973	14.428	9.236	106.803	0.988	9.236
E-GEOD-31979	breast	test	6	0.0	50.0	2.687	10.657	165.011	0.039	-10.657	5.061	31.931	-0.703	-3.362
E-GEOD-32146	colon	test	4	0.5	14.5	5.624	2.153	39.851	0.149	0.088	2.954	22.569	0.607	2.065
E-GEOD-32149	blood whole	test	8	0.375	11.0	12.023	1.338	3.163	0.99	0.838	1.119	1.347	0.996	-0.415
E-GEOD-32396	blood wbc	test	12	0.0	54.5	6.764	5.153	95.113	0.487	4.142	2.21	55.58	0.324	-1.245
E-GEOD-32867	lung	test	24	0.125	69.0	10.458	3.8	17.362	0.924	-1.695	3.252	28.142	0.937	3.252
E-GEOD-34639	blood cd4+ t cell	test	20	0.9	0.0	0.497	0.132	0.122	0.876	-0.111	0.184	0.065	0.921	0.139
E-GEOD-36054	blood leukocyte	test	54	0.593	3.125	4.606	0.64	3.215	0.928	0.244	0.729	1.813	0.971	0.671
E-GEOD-36194	brain cerebellum; brain frontal cortex	test	289	0.433	48.0	27.911	5.675	133.641	0.915	-1.29	3.286	83.059	0.946	-0.557
E-GEOD-39004	breast	test	4	0.0	44.0	5.895	11.126	256.853	0.573	-11.126	4.241	212.246	0.091	-4.241
E-GEOD-40279	blood whole	test	263	0.483	65.0	14.127	3.823	35.396	0.921	1.481	3.725	34.948	0.924	2.213
E-GEOD-41169	blood whole	test	14	0.571	27.0	7.007	1.49	17.904	0.915	-0.822	1.258	4.242	0.957	-0.268
E-GEOD-41826	brain frontal cortex	test	31	0.484	35.0	15.946	2.355	24.96	0.967	-1.194	2.004	10.944	0.979	-0.312
E-GEOD-42700	buccal	test	22	0.5	0.0	0.737	0.779	1.376	0.745	-0.779	0.241	0.378	0.667	0.08
E-GEOD-43256	placenta	test	4	0.5	-0.585	0.041	0.196	0.122	0.837	-0.196	0.086	0.016	0.844	-0.086
E-GEOD-44712	placenta	test	16	0.688	-0.148	0.071	0.305	0.151	0.336	-0.305	0.318	0.104	0.24	0.318
E-GEOD-44763	blood whole	test	9	0.0	58.0	9.333	3.198	24.16	0.876	-0.426	2.599	17.972	0.938	-2.599
E-GEOD-45461	bone marrow	test	9	0.0	-0.345	0.0	0.088	0.015		0.088	0.043	0.004		0.043
E-GEOD-47513	adipose	test	5	0.0	29.0	3.286	14.587	221.725	0.142	-14.587	3.538	19.985	0.235	-3.538
E-GEOD-48325	liver	test	8	0.0	52.0	11.851	4.853	40.963	0.98	4.853	3.767	31.203	0.956	3.767
E-GEOD-48988	colon	test	68	0.0	66.0	9.367	4.31	39.826	0.788	-3.114	2.558	17.385	0.906	-0.443
E-GEOD-49064	blood pbmc	test	4	1.0	58.0	9.083	3.611	13.715	0.94	1.399	1.459	4.714	0.979	-0.611
E-GEOD-49149	pancreas	test	12	0.667	70.0	9.157	7.103	135.514	0.562	7.103	2.829	39.593	0.756	-0.082
E-GEOD-49393	brain prefrontal cortex	test	10	0.7	61.0	8.638	6.509	47.974	0.75	6.194	8.05	67.084	0.47	3.914
E-GEOD-50498	muscle	test	20	1.0	23.0	26.884	14.78	302.025	0.933	-9.847	2.638	21.308	0.989	1.207
E-GEOD-50660	blood whole	test	177	0.695	57.0	6.715	2.643	18.399	0.8	-0.14	2.439	15.276	0.836	1.012
E-GEOD-50759	buccal	test	20	0.4	11.5	7.201	4.954	41.501	0.93	4.954	2.559	18.087	0.84	1.451
E-GEOD-51032	blood buffy coat	test	338	0.222	54.175	7.154	2.836	22.837	0.792	0.279	3.004	18.783	0.816	0.886
E-GEOD-51388	blood whole	test	24	0.625	31.5	10.661	8.292	99.261	0.901	-8.292	1.149	10.593	0.963	0.84
E-GEOD-51954	dermis; epidermis	test	15	0.4	34.0	25.267	11.595	154.817	0.942	-10.258	2.478	26.81	0.989	1.317
E-GEOD-52068	nasopharyngeal	test	10	0.6	43.5	8.879	6.913	70.906	0.867	-6.913	4.456	34.646	0.78	0.333
E-GEOD-52588	blood whole	test	24	0.125	34.0	18.156	7.583	87.438	0.957	-7.044	2.873	21.272	0.972	-0.306
E-GEOD-53128	blood whole	test	18	0.0	63.606	8.063	7.66	123.121	0.576	7.66	4.077	89.573	0.524	4.077
E-GEOD-53162	brain cerebellum; brain prefrontal cortex; brain temporal cortex	test	9	0.889	26.0	15.235	9.302	69.401	0.948	-9.302	5.503	30.25	0.934	-3.588
E-GEOD-53740	blood whole	test	78	0.333	69.5	11.106	8.24	133.27	0.778	8.24	3.678	48.531	0.829	2.232
E-GEOD-54211	buccal	test	9	0.0	55.0	4.522	5.396	71.613	0.254	4.793	2.527	69.527	0.264	-1.557
E-GEOD-54399	blood cord; placenta	test	18	0.667	0.0	0.0	0.207	0.113		-0.207	0.224	0.076		0.224
E-GEOD-54690	blood whole	test	8	1.0	43.5	6.518	4.186	28.562	0.903	-4.186	2.082	8.522	0.954	0.441
E-GEOD-55438	placenta	test	20	0.0	-0.397	0.247	0.084	0.048	0.811	0.004	0.166	0.043	0.589	0.035
E-GEOD-56342	bronchiole	test	10	0.5	63.5	5.426	11.322	134.61	0.695	11.322	4.502	29.885	0.332	0.896
E-GEOD-56515	adrenal; amnion; muscle; pancreas	test	14	0.0	-0.422	0.097	0.834	1.937	0.635	-0.834	0.203	0.471	0.598	-0.203
E-GEOD-56553	blood pbmc	test	15	0.333	26.1	8.104	4.513	28.953	0.96	4.513	1.5	2.76	0.989	0.791
E-GEOD-57484	blood whole	test	5	1.0	10.43	0.178	1.061	10.228	-0.651	-1.061	2.981	7.914	0.04	-2.981
E-GEOD-57767	placenta	test	14	0.0	0.0	0.0	0.178	0.179		-0.149	0.368	0.127		0.368
E-GEOD-58045	blood whole	test	69	0.0	59.904	7.756	3.412	24.473	0.826	1.983	1.961	15.566	0.863	-0.037
E-GEOD-58119	blood serum	test	113	0.0	63.0	6.326	13.986	313.737	0.112	13.535	6.202	60.705	-0.099	1.193
E-GEOD-59457	brain frontal; brain hippocampus; brain midbrain; brain occipital; brain temporal	test	13	0.538	53.0	10.393	9.21	112.635	0.897	9.21	1.403	9.568	0.971	-0.324
E-GEOD-59509	blood whole; saliva; vaginal swab	test	11	0.727	38.0	13.514	3.703	17.94	0.961	2.301	1.137	10.069	0.974	-0.195
E-GEOD-59592	blood whole	test	48	0.562	0.375	0.0	0.617	0.937		-0.617	0.448	0.223		0.448
E-GEOD-61107	brain frontal cortex	test	10	0.8	75.0	9.47	15.009	272.862	0.85	15.009	5.837	60.882	0.878	5.837
E-GEOD-61257	adipose	test	13	0.385	45.0	12.749	6.313	52.137	0.962	-6.313	1.906	12.735	0.964	0.991
E-GEOD-61258	liver	test	32	0.5	54.0	16.165	6.3	89.32	0.872	-0.466	2.992	36.508	0.936	0.545
E-GEOD-61259	muscle	test	11	0.364	48.0	8.202	4.06	23.625	0.808	0.256	4.512	36.155	0.844	4.13
E-GEOD-61380	brain frontal cortex	test	6	0.667	36.5	11.776	4.541	42.262	0.841	-2.183	3.66	23.521	0.925	-1.087
E-GEOD-61454	adipose; liver; muscle	test	108	0.231	42.0	9.581	6.386	66.428	0.764	-5.363	2.964	23.722	0.865	-1.014
E-GEOD-62219	blood leukocyte	test	24	0.0	2.0	1.767	1.589	8.059	0.751	-1.236	0.994	2.411	0.698	0.58
E-GEOD-62867	blood vessel; blood whole	test	8	1.0	53.5	4.635	5.535	48.198	0.796	-3.629	1.942	10.826	0.811	-0.057
E-GEOD-63106	cartilage	test	24	0.417	68.45	8.411	3.603	40.362	0.667	-1.264	3.994	32.735	0.755	-0.822
E-GEOD-63347	brain cerebellum; brain frontal cortex; brain hippocampus; brain midbrain; brain occipital cortex; brain temporal cortex	test	23	0.348	59.0	8.722	10.359	135.815	0.83	10.359	1.235	7.228	0.958	0.837
E-GEOD-64495	blood whole	test	43	0.256	45.1	15.477	2.718	14.73	0.971	1.223	2.223	9.115	0.981	-0.182
E-GEOD-64511	adrenal; bone; bone marrow; brain caudate nucleus; brain cerebellum; brain cingulate gyrus; brain frontal cortex; brain I	test	149	0.349	80.0	25.566	16.078	445.514	0.887	15.893	3.165	54.128	0.963	1.615
E-GEOD-64940	blood cord	test	87	0.54	0.0	0.0	0.121	0.045		0.046	0.249	0.085		0.245
E-GEOD-65638	blood whole	test	7	0.0	25.0	4.435	3.724	108.105	-0.088	-3.459	2.472	52.196	0.079	-0.354
E-GEOD-67024	adipose	test	6	0.0	40.0	12.24	17.474	423.124	0.126	-17.474	10.662	139.09	0.306	-0.311
E-GEOD-67444	blood whole	test	28	0.607	1.0	1.693	1.571	19.836	0.716	-1.571	0.686	6.261	0.706	0.246
E-GEOD-67705	blood whole	test	18	1.0	52.5	11.093	8.773	130.823	0.91	-8.773	2.522	8.922	0.968	-0.409
E-GEOD-71245	blood b cells; blood cd4 t cells; blood cd8 t cells; blood monocyte; blood pbmc	test	10	0.0	49.0	8.149	4.395	90.829	0.304	-2.821	4.811	20.616	0.882	1.637
E-GEOD-71678	placenta	test	132	0.5	0.0	0.0	2.009	74.601		-2.009	0.319	0.106		0.319
E-GEOD-71955	blood cd4 t cells; blood cd8 t cells	test	25	0.04	50.0	9.453	6.14	126.576	0.452	-2.202	3.368	73.278	0.561	0.682
E-GEOD-72338	blood monocyte; blood neutrophil	test	8	0.5	23.0	10.283	1.779	6.349	0.977	-0.46	1.693	4.997	0.987	-1.2
E-GEOD-72556	saliva	test	38	0.447	3.5	0.792	0.517	1.664	0.082	0.413	0.864	1.903	0.207	0.629
E-GEOD-73103	blood whole	test	142	0.556	20.0	4.625	4.114	38.791	0.709	-3.93	1.571	7.421	0.809	-0.291
E-GEOD-73377	placenta	test	15	0.0	0.0	0.0	0.198	0.273		0.037	0.372	0.246		0.297
E-GEOD-73832	intestine	test	12	0.417	61.0	14.683	8.648	177.51	0.743	7.451	7.045	69.895	0.869	-5.169
E-GEOD-74738	placenta	test	12	0.75	-0.024	0.023	0.112	0.029	0.412	0.041	0.419	0.202	0.598	0.419
E-GEOD-75248	placenta	test	130	0.485	0.0	0.0	3.121	10.937		-3.121	0.322	0.105		0.322
E-GEOD-76105	brain superior temporal gyrus	test	14	0.5	78.5	7.832	6.926	62.107	0.799	-6.926	4.141	30.829	0.788	-2.089
E-GEOD-77445	blood whole	test	34	0.588	25.0	15.721	7.702	85.956	0.819	-3.98	4.969	77.989	0.833	-0.859
E-GEOD-77955	colon; liver	test	6	0.833	60.0	11.475	5.546	266.227	0.05	5.452	5.371	139.131	0.355	0.805
E-GEOD-79056	blood cord	test	15	0.4	-0.121	0.096	0.474	0.214	0.686	-0.474	0.091	0.013	0.611	0.086
E-GEOD-83334	blood cord; blood whole	test	12	0.5	5.0	2.357	0.829	1.931	0.88	0.083	0.785	1.339	0.923	0.273
E-MTAB-2344	blood wbc	test	3	0.667	77.0	8.014	5.534	69.766	0.699	-5.277	6.519	35.167	0.706	0.784
E-MTAB-2372	blood wbc	test	19	0.421	49.0	9.831	16.349	302.658	0.869	-16.349	2.497	52.962	0.696	0.043
E-MTAB-487	cd14+ monocytes; cd16+ granulocytes; cd34+ hematopoietic progenitor cells; fibroblasts ; keratinocytes ; mesenchym	test	13	0.0	21.0	13.305	3.869	63.363	0.913	-3.869	3.019	54.677	0.936	-3.019
E-MTAB-625	epidermis	test	12	0.0	45.5	10.332	5.426	66.171	0.749	-4.725	5.496	63.922	0.666	2.781
GSE108213	breast	test	34	0.0	56.5	7.614	11.032	130.355	0.814	-11.032	2.459	13.729	0.883	0.382
GSE115797	skin	test	10	0.7	31.5	9.837	7.94	150.114	0.595	7.713	6.643	70.292	0.699	-3.383
GSE148000	sputum	test	3	0.667	39.0	9.568	5.936	56.361	0.962	-5.936	3.82	16.194	0.95	3.82
GSE15745	brain pons; brain temporal cortex	test	101	0.604	45.0	26.176	3.171	50.708	0.963	-0.663	2.569	47.179	0.965	-0.059
GSE17448	bone marrow	test	7	0.0	25.0	22.173	9.69	100.321	0.915	-9.28	6.862	70.842	0.955	-6.862
GSE19711	blood whole	test	110	0.0	65.0	7.017	4.104	34.882	0.729	2.279	3.103	24.048	0.723	0.697
GSE20236	blood whole	test	38	0.0	63.0	5.295	7.566	75.104	0.667	7.566	3.336	19.249	0.783	3.054
GSE20242	blood cd14+ monocyte; blood cd4+ t cell	test	16	0.312	31.0	13.123	3.06	41.175	0.889	0.669	1.909	24.56	0.954	-1.105
GSE22595	dermis	test	6	0.0	12.5	23.576	10.849	88.372	0.952	-6.406	6.084	74.567	0.974	-6.007
GSE25892	buccal	test	44	0.341	15.0	0.0	1.68	7.717		0.842	0.83	1.988		-0.772
GSE26126	prostate	test	62	1.0	61.5	7.195	5.261	62.832	0.42	0.376	6.121	64.267	0.317	0.607
GSE27317	blood cord	test	68	0.544	0.0	0.0	0.123	0.057		0.059	0.231	0.109		0.216
GSE28746	saliva	test	34	1.0	37.0	8.468	2.437	11.72	0.929	0.501	2.161	8.385	0.942	-0.892
GSE32393	breast	test	10	0.0	50.745	12.669	7.434	88.165	0.697	-0.908	6.061	91.757	0.668	-0.593
GSE34035	saliva	test	79	0.684	28.0	9.064	3.516	38.893	0.807	1.786	2.636	21.472	0.865	-0.719
GSE34257	blood cord	test	34	0.559	0.0	0.369	0.163	0.217	0.528	-0.072	0.37	0.28	0.482	0.36
GSE36166	muscle	test	18	1.0	25.0	0.0	16.457	287.679		-16.457	1.23	54.021		-0.228
GSE36642	blood cord; placenta	test	50	0.48	-0.077	0.033	0.359	21.297	-0.124	-0.359	0.202	0.133	-0.143	0.06
GSE36812	blood cord	test	20	0.0	-0.011	0.018	0.465	112.837	0.088	-0.465	0.17	114.057	0.092	-0.036
GSE37008	blood pbmc	test	38	0.263	33.0	5.144	3.417	18.141	0.745	1.272	3.026	14.951	0.676	-0.232
GSE37988	liver	test	23	0.87	56.0	14.037	4.528	39.334	0.902	-0.756	5.441	46.409	0.885	0.361
GSE38291	muscle	test	5	0.4	65.0	6.524	16.209	325.808	-0.442	16.209	2.798	16.64	0.783	0.644
GSE38608	brain cerebellum; brain occipital cortex	test	7	1.0	8.0	10.335	2.421	28.722	0.982	-2.421	1.003	13.796	0.993	-1.003
GSE38873	cerebellum	test	21	0.0	50.0	7.737	3.732	21.175	0.833	0.992	4.336	27.48	0.741	-1.196
GSE40360	brain frontal lobe	test	8	0.5	71.5	7.952	18.152	335.667	0.731	18.152	4.875	63.177	0.784	4.875
GSE41037	blood whole	test	158	0.538	37.0	17.748	2.624	25.566	0.961	0.769	2.909	19.621	0.969	-0.952
GSE42861	blood leukocyte	test	134	0.313	54.0	10.979	3.572	26.349	0.898	-1.68	3.046	19.814	0.916	0.562
GSE49904	blood buffy coat	test	29	0.276	57.0	14.61	4.659	24.742	0.944	-1.365	3.661	24.276	0.944	-1.679
GSE49905	brain cerebral cortex	test	32	0.656	18.5	14.103	3.242	30.721	0.932	-0.24	3.085	57.223	0.853	-1.562
GSE49907	kidney	test	34	0.676	58.0	10.387	5.548	53.151	0.747	-2.486	3.583	17.6	0.917	-1.258
GSE49908	muscle	test	21	1.0	50.0	16.656	9.052	114.112	0.835	-0.261	4.208	49.371	0.931	-1.406
GSE56606	blood cd14+ monocyte	test	9	0.222	35.0	9.754	2.196	24.022	0.922	-1.67	1.849	16.8	0.951	-1.488

**GSE57285**	blood whole	test	17	0.0	46.0	13.754	7.456	375.678	0.273	5.295	8.85	354.881	0.246	3.522
**GSE59157**	kidney	test	14	0.357	35.0	17.722	24.46	873.669	0.854	24.46	11.201	236.837	0.647	6.943
**GSE59274**	placenta	test	20	0.55	-0.042	0.07	0.327	0.872	-0.384	0.304	0.324	0.927	-0.426	0.313
**GSE63384**	lung	test	14	0.571	63.5	10.727	5.378	34.266	0.86	-2.781	1.936	25.782	0.884	-0.022
**GSE69176**	blood cord	test	61	0.541	0.0	0.0	0.228	29.466		-0.22	0.237	24.487		0.222
**GSE77241**	brain; brain frontal lobe	test	2	1.0	16.5	16.5	0.852	1.044	1.0	0.852	2.686	8.148	1.0	0.966
**GSE90124**	skin	test	129	0.0	60.61	9.359	4.961	49.059	0.793	3.322	2.701	24.641	0.853	0.3
**GSE92767**	saliva	test	22	1.0	49.0	14.116	3.813	20.559	0.963	0.681	2.194	13.752	0.982	1.659
**GSE99624**	blood whole	test	6	0.5	60.5	8.641	2.915	12.734	0.946	-1.955	2.622	12.82	0.945	1.234
**TCGA_BRCA**	breast	test	46	0.0	58.742	15.906	7.931	87.95	0.901	-7.12	4.35	73.691	0.858	0.776
**TCGA_COAD**	colon	test	30	0.633	68.084	14.226	5.389	59.121	0.888	2.898	4.846	50.704	0.906	-0.654
**TCGA_HNSC**	head and neck	test	20	0.85	62.329	11.801	6.507	66.611	0.825	-3.911	3.784	22.139	0.936	-0.73
**TCGA_KIRC**	kidney	test	143	0.657	63.113	11.026	3.624	46.403	0.812	1.637	3.174	34.177	0.857	0.782
**TCGA_KIRP**	kidney	test	20	0.7	63.837	12.938	5.138	55.887	0.884	4.376	3.322	26.077	0.945	2.809
**TCGA_LIHC**	liver	test	19	0.632	65.834	19.426	5.724	140.448	0.871	5.673	3.016	35.235	0.958	-0.107
**TCGA_LUAD**	lung	test	22	0.455	67.173	10.241	5.57	45.686	0.828	-4.063	3.007	18.206	0.909	-1.621
**TCGA_LUSC**	lung	test	28	0.75	71.155	7.708	3.953	26.476	0.747	0.116	2.514	21.976	0.835	1.492
**TCGA_PRAD**	prostate	test	20	1.0	65.962	5.833	3.175	26.514	0.642	2.476	3.247	20.644	0.65	1.231
**TCGA_STAD**	stomach	test	11	0.545	72.723	9.9	4.011	35.755	0.827	-0.623	4.444	29.763	0.84	2.15
**TCGA_THCA**	thyroid	test	23	0.261	47.357	17.372	10.166	121.747	0.942	-10.166	4.719	46.629	0.934	-1.982
**TCGA_UCEC**	uterus	test	13	0.0	62.163	9.84	12.347	295.142	-0.316	-5.249	3.977	81.868	0.449	-0.904

SupplementaryTable4

	deep_pink_importance	deep_pink_importance	deep_pink_importance	deep_pink_importance	shap_importance	shap_importance	shap_importance	shap_importance
	median	mean	std	len	median	mean	std	len
**state_modes**								
**1.0**	3.5065444364502E-15	0.0016862766486502400	0.038308641559258900	9875	2.89386154580517E-07	0.004100140548513980	0.010318695500236500	9875
**2.0**	3.70369221968344E-15	0.004012199854296670	0.08058562598694220	1667	9.3800327688673E-07	0.005426885955420760	0.012228803693853000	1667
**3.0**	3.4376629207338E-15	0.003208798403865520	0.03001565071794000	1242	9.22604635434966E-07	0.008375896643943050	0.018296721293475000	1242
**4.0**	2.47012453951515E-15	0.014532680453407300	0.24960114250072700	517	6.79079513552913E-07	0.007957312826920600	0.021400902100254100	517
**5.0**	3.50255466830605E-15	0.0005814011139496840	0.006958874592984820	156	1.37423954266923E-07	0.003077444624068280	0.008390333320037140	156
**6.0**	2.960868440797E-15	0.0010548753358118600	0.01930653573295280	939	1.1173986928619E-07	0.0035517658531900100	0.01090919102598000	939
**7.0**	1.99788169668087E-15	0.0034063030945771900	0.019567721511338600	35	4.61421091327166E-08	0.0034927299154610700	0.010438484021500100	35
**8.0**	2.01634771074698E-16	2.67524217434576E-13	7.83634489618559E-13	11	9.26004099482457E-08	0.006447126090439150	0.011188357512928400	11
**9.0**	2.19288506050718E-15	0.006783028573025770	0.06087152007246600	120	3.07380394936733E-07	0.004248179128912560	0.011761971998472600	120
**10.0**	2.46292634619127E-15	0.003816839390798710	0.032587542784241900	296	1.42458610871271E-07	0.0029713681987225300	0.009466696042040920	296
**11.0**	2.49085522231763E-15	0.002153816055867650	0.016815068316845500	320	2.23313785173677E-07	0.007723610595825890	0.019912237361682700	320
**12.0**	2.35059795468258E-14	0.007998454470927110	0.03900030157104710	49	0.0016790752602669600	0.0146120718938227	0.03198188759680640	49
**13.0**	2.47118614058762E-14	1.39027209388933E-12	3.15960807278857E-12	29	1.01009739183996E-06	0.004720854791881710	0.010991379143837900	29
**14.0**	4.57059023486112E-15	0.004260424939373100	0.04045102955350080	1390	7.57377186500136E-07	0.007430287651607570	0.016330497384917600	1390
**15.0**	5.70328107752386E-15	0.01046260212757230	0.09161794539709640	278	8.46235268516986E-07	0.008721054031362890	0.021427906671223000	278
**16.0**	7.31441089217093E-15	0.007321801868513640	0.06991368707713090	926	8.41866544267151E-07	0.007592660946796800	0.018260952926027400	926
**17.0**	4.38679477565722E-15	0.0032440594004696900	0.047216031247879600	995	8.761872211062E-08	0.004357338959152660	0.01234476108668150	995
**18.0**	2.94221246178056E-15	0.00133535716171223	0.01801923965168090	2520	8.53497774033219E-08	0.003266349254635280	0.00998204326898277	2520

DATASET DESCRIPTION

TRAIN/VALIDATION/TEST

GSE27317: newborn umbilical cord buffy coat samples. All samples were recorded as having zero age. The platform used was Illumina's Infinium 27k Human Methylation Beadchip. (PMID: 21453505)

GSE41037: whole blood samples in schizophrenia patients and healthy subjects. Non-healthy patient samples were separated for further analysis and not included in training, validation, or testing. The platform used was Illumina's Infinium 27k Human Methylation Beadchip. (PMID: 23034122, 25424713, 26949191)

GSE38873: postmortem cerebellum samples from patients with schizophrenia or bipolar disorder or depression and healthy controls. Non-healthy patient samples were separated for further analysis and not included in training, validation, or testing. Out of 155 samples, there were 13 random replicates. Replicates were separated for further analysis and not included in training, validation, or testing. The platform used was Illumina's Infinium 27k Human Methylation Beadchip. (PMID: 25243493)

GSE15745: postmortem frozen brain samples from the cerebellum, frontal cortex, pons, and temporal cortex of each subject (four samples per subject). The platform used was Illumina's Infinium 27k Human Methylation Beadchip. (PMID: 20485568)

GSE32393: breast samples from women with or without breast cancer. Cancer samples were separated for further analysis and not included in training, validation, or testing. The platform used was Illumina's Infinium 27k Human Methylation Beadchip. (PMID: 22346766)

GSE25892: buccal epithelial cell samples from adolescents whose parents reported high levels of stress during the children's infancy. The platform used was Illumina's Infinium 27k Human Methylation Beadchip. (PMID: 21883162)

GSE20242: CD4+ T-cells and CD14+ monocytes samples. The platform used was Illumina's Infinium 27k Human Methylation Beadchip. (PMID: 20219945)

GSE22595: human dermal fibroblast samples expanded *in vitro*. The platform used was Illumina's Infinium 27k Human Methylation Beadchip. (PMID: 21347436)

GSE37988: liver samples from tumors or adjacent to tumors (two samples per patient). Cancer samples were separated for further analysis and not included in training, validation, or testing. The platform used was Illumina's Infinium 27k Human Methylation Beadchip. (PMID: 22976466)

GSE17448: mesenchymal stromal cells passaged *in vitro*. The platform used was Illumina's Infinium 27k Human Methylation Beadchip. (PMID: 19895632, 25241740)

GSE36642: blood mononuclear cell, human umbilical vascular endothelial cell, and placenta samples from monozygotic and dizygotic twins. Given that gestational week information was available, the age was encoded with the formula shown in Methods. The platform used was Illumina's Infinium 27k Human Methylation Beadchip. (PMID: 22800725)

GSE26126: prostate samples from normal and cancer tissue, and cultured prostate cells overexpressing DNMTs and EZH2. Cultured samples were disregarded. Cancer samples were separated for further analysis and not included in training, validation, or testing. The platform used was Illumina's Infinium 27k Human Methylation Beadchip. (PMID: 21521786)

GSE34035: saliva samples from alcohol users. The platform used was Illumina's Infinium 27k Human Methylation Beadchip. (PMID: 20386599)

GSE28746: saliva samples from pairs of identical twins. The platform used was Illumina's Infinium 27k Human Methylation Beadchip. (PMID: 21731603)

GSE20236: whole blood samples. The platform used was Illumina's Infinium 27k Human Methylation Beadchip. (PMID: 20219945)

GSE19711: whole blood samples from patients with or without ovarian cancer. Subjects with ovarian cancer were disregarded. The platform used was Illumina's Infinium 27k Human Methylation Beadchip. (PMID: 20219944)

GSE37008: peripheral blood mononuclear cell samples. Out of 94 samples, there were five random technical replicates. Replicates were discarded. The platform used was Illumina's Infinium 27k Human Methylation Beadchip. (PMID: 23045638)

GSE36812: blood cord samples from newborns. Given that gestational week information was available, the age was encoded with the formula shown in Methods. The platform used was Illumina's Infinium 27k Human Methylation Beadchip.

GSE34257: blood cord samples and whole blood samples from 9-month-old infants. Blood cord samples were recorded as having zero age. The platform used was Illumina's Infinium 27k Human Methylation Beadchip. (PMID: 22307237)

GSE38608: postmortem brain cerebellum and occipital cortex samples from subjects with autism and healthy controls. Non-healthy patient samples were separated for further analysis and not included in training, validation, or testing. Out of 34 samples, there were three replicates. Replicates were separated for further analysis and not included in training, validation, or testing. The platform used was Illumina's Infinium 27k Human Methylation Beadchip. (PMID: 22984548)

GSE38291: muscle and adipose samples from monozygotic twins discordant for type 2 diabetes. Non-healthy patient samples were separated for further analysis and not included in training, validation, or testing. The platform used was Illumina's Infinium 27k Human Methylation Beadchip. (PMID: 23251491)

GSE36166: muscle samples from subjects receiving a control or a high-fat diet. Both groups were included. The platform used was Illumina's Infinium 27k Human Methylation Beadchip. (PMID: 22961225)

GSE63384: lung samples from tumors or adjacent to tumors (two samples per patient). Cancer samples were separated for further analysis and not included in training, validation, or testing. The platform used was Illumina's Infinium 27k Human Methylation Beadchip. (PMID: 26134223)

GSE59274: placental tissue samples from women with uncomplicated pregnancies and with preeclampsia. Both groups were included. Given that gestational week information was available, the age was encoded with the formula shown in Methods. The platform used was Illumina's Infinium 27k Human Methylation Beadchip. (PMID: 25247495)

GSE57285: whole blood samples from women with BRCA1 wild-type or mutants, and with or without breast cancer. Subjects with cancer were disregarded. BRCA1 mutant patient samples were separated for further analysis and not included in training, validation, or testing. The platform used was Illumina's Infinium 27k Human Methylation Beadchip. (PMID: 25067956)

GSE56606: CD14+ monocyte samples from monozygotic twins discordant for type 1 diabetes. Non-healthy patient samples were separated for further analysis and not included in training, validation, or testing. The platform used was Illumina's Infinium 27k Human Methylation Beadchip. (PMID: 21980303)

GSE49908: skeletal muscle samples. The platform used was Illumina's Infinium 27k Human Methylation Beadchip. (PMID: 24034465)

GSE49907: kidney samples. The platform used was Illumina's Infinium 27k Human Methylation Beadchip. (PMID: 24034465)

GSE49905: brain cerebral cortex samples. The platform used was Illumina's Infinium 27k Human Methylation Beadchip. (PMID: 24034465)

GSE49904: blood buffy coat samples. The platform used was Illumina's Infinium 27k Human Methylation Beadchip. (PMID: 24034465)

E-GEOD-43256: placental chorionic villi from electively terminated pregnancies or miscarriages. Both groups were included. Given that gestational week information was available, the age was encoded with the formula shown in Methods. The platform used was Illumina's Infinium 27k Human Methylation Beadchip. (PMID: 23583422)

E-GEOD-64940: cord blood samples from newborns. All samples were encoded as zero age. The platform used was Illumina's Infinium 27k Human Methylation Beadchip. (PMID: 25742137)

E-MTAB-2344: white blood cell samples in patients with stroke and/or obesity and healthy controls. Non-healthy patient samples were separated for further analysis and not included in training, validation, or testing. The platform used was Illumina's Infinium 27k Human Methylation Beadchip. (PMID: 25429063)

E-GEOD-62867: advanced atherosclerotic plaques, atherosclerotic-resistant internal mammary arteries, and great saphenous veins samples. Non-healthy patient samples were separated for further analysis and not included in training, validation, or testing. The platform used was Illumina's Infinium 27k Human Methylation Beadchip. (PMID: 25856389)

E-GEOD-44763: whole blood samples from obese and lean subjects. Non-healthy patient samples were separated for further analysis and not included in training, validation, or testing. The platform used was Illumina's Infinium 27k Human Methylation Beadchip. (PMID: 25010727)

E-GEOD-48988: colon samples from subjects with different lifestyle factors. The platform used was Illumina's Infinium 27k Human Methylation Beadchip. (PMID: 24973978)

E-GEOD-58119: serum samples from healthy postmenopausal women. The platform used was Illumina's Infinium 27k Human Methylation Beadchip. (PMID: 25067956)

E-GEOD-58045: whole blood samples. The platform used was Illumina's Infinium 27k Human Methylation Beadchip. (PMID: 22532803)

E-GEOD-57484: whole blood samples from normal and obese children. Non-healthy patient samples were separated for further analysis and not included in training, validation, or testing. The platform used was Illumina's Infinium 27k Human Methylation Beadchip.

E-GEOD-54211: buccal samples from oral lichenoid disease patients and healthy controls. Non-healthy patient samples were not included in training, validation, or testing. The platform used was Illumina's Infinium 27k Human Methylation Beadchip.

E-GEOD-56342: small airway bronchioles samples from chronic obstructive pulmonary disease and healthy controls. Non-healthy patient samples were separated for further analysis and not included in training, validation, or testing. The platform used was Illumina's Infinium 27k Human Methylation Beadchip. (PMID: 24298892)

E-GEOD-27044: peripheral blood leukocytes from autistic children and unaffected siblings. Non-healthy patient samples were separated for further analysis and not included in training, validation, or testing. The platform used was Illumina's Infinium 27k Human Methylation Beadchip. (PMID: 22300631)

E-GEOD-36194: postmortem cerebellum and frontal cortex samples (two samples per subject). The platform used was Illumina's Infinium 27k Human Methylation Beadchip.

E-GEOD-21232: pancreatic islet samples from patients with type 2 diabetes and healthy controls. Non-healthy patient samples were separated for further analysis and not included in training, validation, or testing. The platform used was Illumina's Infinium 27k Human Methylation Beadchip. (PMID: 22293752)

E-GEOD-32867: lung tumor and adjacent non-tumor lung samples. Cancer samples were separated for further analysis and not included in training, validation, or testing. The platform used was Illumina's Infinium 27k Human Methylation Beadchip. (PMID: 22613842)

E-GEOD-30759: uterine cervix and cervical cancer samples. Cancer samples were separated for further analysis and not included in training, validation, or testing. The platform used was Illumina's Infinium 27k Human Methylation Beadchip. (PMID: 22346766, 22453031, 27103033)

E-GEOD-30758: uterine cervix samples. The platform used was Illumina's Infinium 27k Human Methylation Beadchip. (PMID: 22346766, 22453031, 27103033)

E-GEOD-32396: whole blood sample from women with or without BRCA1 mutation, with or without breast cancer. Cancer samples were separated for further analysis and not included in training, validation, or testing. The platform used was Illumina's Infinium 27k Human Methylation Beadchip. (PMID: 22346766)

E-GEOD-31979: breast tumor and adjacent non-tumor breast samples. Cancer samples were separated for further analysis and not included in training, validation, or testing. The platform used was Illumina's Infinium 27k Human Methylation Beadchip. (PMID: 21825015)

E-MTAB-625: epidermis samples. The platform used was Illumina's Infinium 27k Human Methylation Beadchip. (PMID: 21993563)

E-MTAB-487: CD34+ hematopoietic progenitor cell, CD16+ granulocytes, CD14+ monocytes, mesenchymal stem cells, keratinocytes, and fibroblast samples. The platform used was Illumina's Infinium 27k Human Methylation Beadchip. (PMID: 21427290)

GSE90124: skin peri-umbilical punch samples. The platform used was Illumina's Infinium 450k Human Methylation Beadchip. (PMID: 27993549)

GSE115797: skin samples from psoriatic skin disease and adjacent normal skin samples. Non-healthy patient samples were separated for further analysis and not included in training, validation, or testing. The platform used was Illumina's Infinium 450k Human Methylation Beadchip. (PMID: 30092825)

GSE99624: whole blood samples from osteoporotic and healthy control patients. Non-healthy patient samples were separated for further analysis and not included in training, validation, or testing. The platform used was Illumina's Infinium 450k Human Methylation Beadchip. (PMID: 28926142)

GSE108213: breast samples. The platform used was Illumina's Infinium 450k Human Methylation Beadchip. (PMID: 30821641)

GSE92767: saliva samples. The platform used was Illumina's Infinium 450k Human Methylation Beadchip. (PMID: 28419903)

GSE69176: umbilical cord blood samples from newborns. All ages were encoded as zero age. The platform used was Illumina's Infinium 450k Human Methylation Beadchip.

GSE40360: postmortem frontal lobe white matter in multiple sclerosis patients and healthy controls. Non-healthy patient samples were separated for further analysis and not included in training, validation, or testing. The platform used was Illumina's Infinium 450k Human Methylation Beadchip. (PMID: 24270187)

GSE59157: kidney samples from tumor patients and healthy subjects. Cancer samples were separated for further analysis and not included in training, validation, or testing. The platform used was Illumina's Infinium 450k Human Methylation Beadchip. (PMID: 25134821)

GSE42861: peripheral blood leukocytes from rheumatoid arthritis patients and healthy controls. Samples from rheumatoid arthritis patients were discarded. The platform used was Illumina's Infinium 450k Human Methylation Beadchip. (PMID: 29921915)

E-MTAB-2372: white blood cell samples from obese patients subject to two different diets. Both groups were included. The platform used was Illumina's Infinium 450k Human Methylation Beadchip.

E-GEOD-73832: intestinal samples from patients with small intestinal neuroendocrine tumors and healthy controls. Samples from patients with cancer were not included in training, validation, or testing. The platform used was Illumina's Infinium 450k Human Methylation Beadchip. (PMID: 26169971, 28049633)

E-GEOD-71678: placental tissue samples exposed to different levels of arsenic. All groups were included. All ages were encoded as zero age. The platform used was Illumina's Infinium 450k Human Methylation Beadchip. (PMID: 26771251, 29373860)

E-GEOD-71245: blood samples from different types of blood cells. The platform used was Illumina's Infinium 450k Human Methylation Beadchip. (PMID: 26291385)

E-GEOD-83334: whole blood and cord blood from newborns and infants measured longitudinally. The platform used was Illumina's Infinium 450k Human Methylation Beadchip. (PMID: 27259700)

E-GEOD-75248: placental samples from newborns. All ages were encoded as zero age. The platform used was Illumina's Infinium 450k Human Methylation Beadchip. (PMID: 27366929, 29373860, 28621618)

E-GEOD-77955: liver, colon, and small intestine samples from patients with and without cancer. Cancer samples were separated for further analysis and not included in training, validation, or testing. The platform used was Illumina's Infinium 450k Human Methylation Beadchip. (PMID: 27270421)

E-GEOD-67705: blood samples from HIV+ and HIV- subjects. Non-healthy patient samples were separated for further analysis and not included in training, validation, or testing. The platform used was Illumina's Infinium 450k Human Methylation Beadchip. (PMID: 27105112)

E-GEOD-77445: whole blood samples from subjects with different stress levels. All groups were included. The platform used was Illumina's Infinium 450k Human Methylation Beadchip. (PMID: 26997371)

E-GEOD-79056: cord blood samples. Given that gestational week information was available, the age was encoded with the formula shown in Methods. The platform used was Illumina's Infinium 450k Human Methylation Beadchip.

E-GEOD-72556: saliva samples from children. The platform used was Illumina's Infinium 450k Human Methylation Beadchip.

E-GEOD-52068: nasopharyngeal epithelial tissue from tumors or healthy controls. Cancer samples were separated for further analysis and not included in training, validation, or testing. The platform used was Illumina's Infinium 450k Human Methylation Beadchip. (PMID: 26443805, 28146149)

E-GEOD-74738: placental chorionic villi samples with control pregnancies and several disorders. Non-healthy patient samples were separated for further analysis and not included in training, validation, or testing. Samples from maternal tissue were excluded. The platform used was Illumina's Infinium 450k Human Methylation Beadchip. (PMID: 26769960)

E-GEOD-76105: postmortem brain superior temporal gyrus of Alzheimer's disease and control subjects. Non-healthy patient samples were not included in training, validation, or testing. The platform used was Illumina's Infinium 450k Human Methylation Beadchip. (PMID: 26769960)

E-GEOD-65638: whole blood samples twins. The platform used was Illumina’s Infinium 450k Human Methylation Beadchip. (PMID: 26635134)

E-GEOD-71955: CD4+ and CD8+ T-cell samples from subjects with Graves’ disease or healthy controls. Non-healthy patient samples were separated for further analysis and not included in training, validation, or testing. The platform used was Illumina’s Infinium 450k Human Methylation Beadchip. (PMID: 26459776)

E-GEOD-63106: knee and hip cartilage from patients who underwent joint replacement surgery primarily due to osteoarthritis. The platform used was Illumina’s Infinium 450k Human Methylation Beadchip.

E-GEOD-73377: placental tissue samples from healthy and preeclamptic women. Both groups were included. Given that gestational week information was available, the age was encoded with the formula shown in Methods. The platform used was Illumina’s Infinium 450k Human Methylation Beadchip. (PMID: 26510177)

E-GEOD-56515: muscle, amnion, pancreas, and adrenal gland fetal tissues. Given that gestational week information was available, the age was encoded with the formula shown in Methods. The platform used was Illumina’s Infinium 450k Human Methylation Beadchip. (PMID: 26492326)

E-GEOD-73103: whole blood samples. The platform used was Illumina’s Infinium 450k Human Methylation Beadchip. (PMID: 26449484)

E-GEOD-67024: abdominal subcutaneous fat cells from obese and healthy subjects. Non-healthy patient samples were separated for further analysis and not included in training, validation, or testing. The platform used was Illumina’s Infinium 450k Human Methylation Beadchip. (PMID: 26351548)

E-GEOD-72338: neutrophils and monocytes from patients with tuberculosis and household controls. Non-healthy patient samples were separated for further analysis and not included in training, validation, or testing. The platform used was Illumina’s Infinium 450k Human Methylation Beadchip. (PMID: 26351548)

E-GEOD-59457: various postmortem brain region tissue samples from HIV+ and HIV- subjects. Non-healthy patient samples were separated for further analysis and not included in training, validation, or testing. The platform used was Illumina’s Infinium 450k Human Methylation Beadchip. (PMID: 25969563)

E-GEOD-64511: human bone, brain tissue samples, and 30 tissues from a 112-year-old woman. The platform used was Illumina’s Infinium 450k Human Methylation Beadchip. (PMID: 26000617)

E-GEOD-64495: whole blood from subjects with or without developmental disorder syndrome X. Non-healthy patient samples were separated for further analysis and not included in training, validation, or testing. The platform used was Illumina’s Infinium 450k Human Methylation Beadchip. (PMID: 25991677)

E-GEOD-59509: whole blood, saliva, menstrual blood, vaginal swab, and semen samples. Semen samples were not included. The platform used was Illumina’s Infinium 450k Human Methylation Beadchip. (PMID: 25796047)

E-GEOD-67444: neonatal blood samples. All ages were encoded as zero age. The platform used was Illumina’s Infinium 450k Human Methylation Beadchip.

E-GEOD-62219: longitudinal peripheral blood leukocyte samples from infants. The platform used was Illumina's Infinium 450k Human Methylation Beadchip. (PMID: 25874017)

E-GEOD-51954: epidermal and dermal samples from sun-exposed and sun-protected body sites. Sun-exposed patient samples were separated for further analysis and not included in training, validation, or testing. The platform used was Illumina's Infinium 450k Human Methylation Beadchip. (PMID: 25886480)

E-GEOD-52588: blood samples from subjects with or without Down syndrome. Non-healthy patient samples were separated for further analysis and not included in training, validation, or testing. The platform used was Illumina's Infinium 450k Human Methylation Beadchip. (PMID: 25701644, 25678027, 25701668)

E-GEOD-36054: peripheral blood leukocytes from children. The platform used was Illumina's Infinium 450k Human Methylation Beadchip. (PMID: 22300631)

E-GEOD-50660: peripheral blood from never, former, and current smokers. Current smokers' samples were separated for further analysis and not included in training, validation, or testing. The platform used was Illumina's Infinium 450k Human Methylation Beadchip. (PMID: 25424692)

E-GEOD-61259: muscle samples. The platform used was Illumina's Infinium 450k Human Methylation Beadchip. (PMID: 25313081)

E-GEOD-61258: liver samples. The platform used was Illumina's Infinium 450k Human Methylation Beadchip. (PMID: 25313081)

E-GEOD-61257: adipose samples. The platform used was Illumina's Infinium 450k Human Methylation Beadchip. (PMID: 25313081)

E-GEOD-61454: liver, muscle, and subcutaneous adipose tissue samples. The platform used was Illumina's Infinium 450k Human Methylation Beadchip. (PMID: 25282492)

E-GEOD-61380: postmortem prefrontal cortex brain samples from subjects with schizophrenia and controls. Non-healthy patient samples were separated for further analysis and not included in training, validation, or testing. The platform used was Illumina's Infinium 450k Human Methylation Beadchip. (PMID: 26351548)

E-GEOD-54690: blood samples from subjects with or without dietary flavanol supplementation. Both groups were included. The platform used was Illumina's Infinium 450k Human Methylation Beadchip. (PMID: 24763279)

E-GEOD-49149: pancreatic ductal adenocarcinoma samples and normal adjacent tissues. Cancer samples were separated for further analysis and not included in training, validation, or testing. The platform used was Illumina's Infinium 450k Human Methylation Beadchip. (PMID: 24500968, 26909576)

E-GEOD-55438: placental samples from the first and third trimesters. They were encoded as being one month and half and seven months and a half into the pregnancy, respectively; the age was encoded with

the formula shown in Methods. The platform used was Illumina's Infinium 450k Human Methylation Beadchip. (PMID: 24951174)

E-GEOD-61107: postmortem brain samples from subjects with schizophrenia and controls. Non-healthy patient samples were separated for further analysis and not included in training, validation, or testing. The platform used was Illumina's Infinium 450k Human Methylation Beadchip. (PMID: 24399042)

E-GEOD-53740: blood samples from progressive supranuclear palsy patients, frontotemporal dementia patients, and healthy controls. Non-healthy patient samples were separated for further analysis and not included in training, validation, or testing. The platform used was Illumina's Infinium 450k Human Methylation Beadchip. (PMID: 24603599, 25589773)

E-GEOD-57767: placental tissue samples from term and preeclampsia pregnancies. Only normal pregnancies were included with zero age, as the other samples did not have gestational week. The platform used was Illumina's Infinium 450k Human Methylation Beadchip. (PMID: 24963923)

E-GEOD-49064: peripheral blood mononucleated cells cultured in vitro with or without the synthetic transcription modulating agent WY14,643. Samples cultured with WY14,643 were removed. The platform used was Illumina's Infinium 450k Human Methylation Beadchip. (PMID: 24789080)

E-GEOD-50759: ectodermal cells of autism disorder patients and healthy controls. Non-healthy patient samples were separated for further analysis and not included in training, validation, or testing. The platform used was Illumina's Infinium 450k Human Methylation Beadchip. (PMID: 24875834)

E-GEOD-56553: peripheral mononuclear blood cells of asthmatic patients. The platform used was Illumina's Infinium 450k Human Methylation Beadchip. (PMID: 24963923)

E-GEOD-54446: umbilical cord samples. All ages were encoded as zero age. The platform used was Illumina's Infinium 450k Human Methylation Beadchip. (PMID: 24709820)

E-GEOD-54399: cord blood and placenta. Samples C1 and C20 were contaminated and were not included. All ages were encoded as zero age. The platform used was Illumina's Infinium 450k Human Methylation Beadchip. (PMID: 29017633, 25043696, 28680507)

E-GEOD-53162: postmortem brain samples from autism cases and controls. Non-healthy patient samples were separated for further analysis and not included in training, validation, or testing. The platform used was Illumina's Infinium 450k Human Methylation Beadchip. (PMID: 23999529)

E-GEOD-53128: whole blood samples. The platform used was Illumina's Infinium 450k Human Methylation Beadchip. (PMID: 24496475)

E-GEOD-50498: muscle samples. The platform used was Illumina's Infinium 450k Human Methylation Beadchip. (PMID: 24304487)

E-GEOD-47513: subcutaneous abdominal and gluteal adipose tissue samples. The platform used was Illumina's Infinium 450k Human Methylation Beadchip. (PMID: 24340035)

E-GEOD-49393: postmortem prefrontal cortex tissues of individuals with alcohol use disorders or controls. Non-healthy patient samples were separated for further analysis and not included in training, validation, or testing. The platform used was Illumina's Infinium 450k Human Methylation Beadchip. (PMID: 24163133, 26763658)

E-GEOD-39004: breast tissue samples with or without tumors. Cancer samples were separated for further analysis and not included in training, validation, or testing. The platform used was Illumina's Infinium 450k Human Methylation Beadchip. (PMID: 24316975, 30501643, 25016594)

E-GEOD-51388: blood samples taken longitudinally from monozygotic twins. The platform used was Illumina's Infinium 450k Human Methylation Beadchip. (PMID: 26248206)

E-GEOD-51032: blood buffy coat. The platform used was Illumina's Infinium 450k Human Methylation Beadchip.

E-GEOD-48325: liver samples from patients with non-alcoholic fatty liver disease and healthy controls. Non-healthy patient samples were separated for further analysis and not included in training, validation, or testing. The platform used was Illumina's Infinium 450k Human Methylation Beadchip. (PMID: 23931760, 25313081)

E-GEOD-44712: placental tissue from normal and preeclamptic pregnancies. Both groups were included. Given that gestational week information was available, the age was encoded with the formula shown in Methods. The platform used was Illumina's Infinium 450k Human Methylation Beadchip. (PMID: 23770704)

E-GEOD-45461: B cell precursor cells from 20-24 weeks of gestation. Age was encoded as 22 gestational weeks and transformed with the formula shown in Methods. The platform used was Illumina's Infinium 450k Human Methylation Beadchip. (PMID: 23074194)

E-GEOD-40279: whole blood samples. The platform used was Illumina's Infinium 450k Human Methylation Beadchip. (PMID: 23177740)

E-GEOD-41169: whole blood of schizophrenia and healthy subjects. Non-healthy patient samples were separated for further analysis and not included in training, validation, or testing. The platform used was Illumina's Infinium 450k Human Methylation Beadchip. (PMID: 25969563)

E-GEOD-32149: peripheral blood from ulcerative colitis, Crohn's disease, and healthy subjects. Non-healthy patient samples were separated for further analysis and not included in training, validation, or testing. The platform used was Illumina's Infinium 450k Human Methylation Beadchip.

E-GEOD-41826: brain frontal cortex samples from depression and healthy subjects. Non-healthy patient samples were separated for further analysis and not included in training, validation, or testing. The platform used was Illumina's Infinium 450k Human Methylation Beadchip. (PMID: 23426267)

E-GEOD-42700: buccal samples from monozygotic and dizygotic twins at birth and at 18 months of age. The platform used was Illumina's Infinium 450k Human Methylation Beadchip. (PMID: 23697701)

E-GEOD-32146: normal, Crohn's disease, and ulcerative colitis colon mucosa. Non-healthy patient samples were separated for further analysis and not included in training, validation, or testing. The platform used was Illumina's Infinium 450k Human Methylation Beadchip.

E-GEOD-30870: cord blood and whole blood from newborns and nonagenarians. Age from newborns was encoded as zero age. The platform used was Illumina's Infinium 450k Human Methylation Beadchip. (PMID: 26161907)

E-GEOD-34639: CD4+ T-cells from subjects with or without dietary allergies. Both groups were included. The platform used was Illumina's Infinium 450k Human Methylation Beadchip. (PMID: 22495533, 24762976)

E-GEOD-63347: various brain samples from patients with or without autism. Non-healthy patient samples were separated for further analysis and not included in training, validation, or testing. The platform used was Illumina's Infinium 450k Human Methylation Beadchip. (PMID: 25678027)

E-GEOD-59592: blood from infants exposed to varying degrees of aflatoxin B1. All groups were included. The platform used was Illumina's Infinium 450k Human Methylation Beadchip. (PMID: 25855716, 26062908, 29034560)

GSE77241: astrocytoma, fetal brain, and adult brain tissue samples. Cancer samples were disregarded. The platform used was Illumina's Infinium 450k Human Methylation Beadchip. (PMID: 27229157)

GSE148000: sputum samples from subjects with asthma, COPD, and healthy controls. Non-healthy patient samples were separated for further analysis and not included in training, validation, or testing. The platform used was Illumina's Infinium 450k Human Methylation Beadchip. (PMID: 33076907)

TCGA-LUSC: bronchus and lung, normal and cancer. Cancer samples were not included in training, validation, or testing.

TCGA-THCA: thyroid gland, normal and cancer. Cancer samples were not included in training, validation, or testing.

TCGA-HNSC: head and neck, normal and cancer. Cancer samples were not included in training, validation, or testing.

TCGA-KIRC: kidney, normal and cancer. Cancer samples were not included in training, validation, or testing.

TCGA-KIRP: kidney, normal and cancer. Cancer samples were not included in training, validation, or testing.

TCGA-LUAD: bronchus and lung, normal and cancer. Cancer samples were not included in training, validation, or testing.

TCGA-PRAD: prostate, normal and cancer. Cancer samples were not included in training, validation, or testing.

TCGA-STAD: stomach, normal and cancer. Cancer samples were not included in training, validation, or testing.

TCGA-COAD: colon, normal and cancer. Cancer samples were not included in training, validation, or testing.

TCGA-LIHC: liver and intrahepatic bile ducts, normal and cancer. Cancer samples were not included in training, validation, or testing.

TCGA-UCEC: uterus, normal and cancer. Cancer samples were not included in training, validation, or testing.

TCGA-BRCA: breast, normal and cancer. Cancer samples were not included in training, validation, or testing.

RELIABILITY

GSE55763: whole blood from 2,664 human samples and 36 technical replicates. Only the 36 duplicates were included for the analysis of reliability. The platform used was Illumina's Infinium 450k Human Methylation Beadchip. (PMID: 25853392, 28002404)

IN VITRO

GSE91069: 31 samples with different groups of transformation and senescent cells. Fibroblasts were subdivided in early passage, near senescence, oncogene-induced senescence, and replicative senescence. Only these four groups were used for analysis. The platform used was Illumina's Infinium 450k Human Methylation Beadchip. (PMID: 29438699)

GSE30653: hESCs and hiPSC samples representing over 100 cell lines. The platform used was Illumina's Infinium 27k Human Methylation Beadchip. (PMID: 22560082)

GSE100249: HEK293 cells with no mtDNA from a dominant negative mitochondrial DNA polymerase construct and 143B rho0 and rho+ chronically depleted of mtDNA due to ethidium bromide. Only 143B were analyzed. The platform used was Illumina's Infinium 27k Human Methylation Beadchip. (PMID: 29668680, 30737248)

GSE45727: H9 hESCs with or without NLRP7 knockdown, both in undifferentiated and BMP4-induced states. The platform used was Illumina's Infinium 27k Human Methylation Beadchip. (PMID: 24105472)

GSE142439: human fibroblasts and endothelial cells with transient expression of OCT4, SOX2, KLF4, c-MYC, LIN28 and NANOG. The platform used was Illumina's Infinium MethylationEPIC. (PMID: 32210226)

GSE54848: reprogramming time course from human fibroblasts to iPSCs with OSKM. The platform used was Illumina's Infinium 27k Human Methylation Beadchip. (PMID: 25097266)

CANCER

GSE32393: breast samples from women with or without breast cancer. Cancer samples were used for the cancer comparison whereas healthy samples were used earlier for train/validation/test. The platform used was Illumina's Infinium 27k Human Methylation Beadchip. (PMID: 22346766)

GSE37988: liver samples from tumors or adjacent to tumors (two samples per patient). Cancer samples were used for the cancer comparison whereas healthy samples were used earlier for train/validation/test. The platform used was Illumina's Infinium 27k Human Methylation Beadchip. (PMID: 22976466)

GSE26126: prostate samples from normal and cancer tissue, and cultured prostate cells overexpressing DNMTs and EZH2. Cancer samples were used for the cancer comparison whereas healthy samples were used earlier for train/validation/test. The platform used was Illumina's Infinium 27k Human Methylation Beadchip. (PMID: 21521786)

GSE63384: lung samples from tumors or adjacent to tumors (two samples per patient). Cancer samples were used for the cancer comparison whereas healthy samples were used earlier for train/validation/test. The platform used was Illumina's Infinium 27k Human Methylation Beadchip. (PMID: 26134223)

GSE59157: kidney samples from tumor patients and healthy subjects. Cancer samples were used for the cancer comparison whereas healthy samples were used earlier for train/validation/test. The platform used was Illumina's Infinium 450k Human Methylation Beadchip. (PMID: 25134821)

E-GEOD-32867: lung tumor and adjacent non-tumor lung samples. Cancer samples were used for the cancer comparison whereas healthy samples were used earlier for train/validation/test. The platform used was Illumina's Infinium 27k Human Methylation Beadchip. (PMID: 22613842)

E-GEOD-30759: uterine cervix and cervical cancer samples. Cancer samples were used for the cancer comparison whereas healthy samples were used earlier for train/validation/test. The platform used was Illumina's Infinium 27k Human Methylation Beadchip. (PMID: 22346766, 22453031, 27103033)

E-GEOD-31979: breast tumor and adjacent non-tumor breast samples. Cancer samples were used for the cancer comparison whereas healthy samples were used earlier for train/validation/test. The platform used was Illumina's Infinium 27k Human Methylation Beadchip. (PMID: 21825015)

E-GEOD-77955: liver, colon, and small intestine samples from patients with and without cancer. Cancer samples were used for the cancer comparison whereas healthy samples were used earlier for train/validation/test. The platform used was Illumina's Infinium 450k Human Methylation Beadchip. (PMID: 27270421)

E-GEOD-52068: nasopharyngeal epithelial tissue from tumors or healthy controls. Cancer samples were used for the cancer comparison whereas healthy samples were used earlier for train/validation/test. The platform used was Illumina's Infinium 450k Human Methylation Beadchip. (PMID: 26443805, 28146149)

E-GEOD-49149: pancreatic ductal adenocarcinoma samples and normal adjacent tissues. Cancer samples were used for the cancer comparison whereas healthy samples were used earlier for train/validation/test.

The platform used was Illumina's Infinium 450k Human Methylation Beadchip. (PMID: 24500968, 26909576)

E-GEOD-39004: breast tissue samples with or without tumors. Cancer samples were used for the cancer comparison whereas healthy samples were used earlier for train/validation/test. The platform used was Illumina's Infinium 450k Human Methylation Beadchip. (PMID: 24316975, 30501643, 25016594)

GSE53051: samples from normal tissue and lung, pancreas, colon, thyroid, and breast cancer. Samples were used for the cancer comparison. The platform used was Illumina's Infinium 450k Human Methylation Beadchip. (PMID: 25191524)
