# Supplementary material for: A pan-tissue DNA-methylation epigenetic clock based on deep learning
Source: NPJ Aging. 2022 Apr 19;8(1):4. doi: 10.1038/s41514-022-00085-y (PMC9158789; doi:10.1038/s41514-022-00085-y)
Supplement: Supplementary file 2 — Supplementary Information [file 41514_2022_85_MOESM2_ESM.pdf]

Supplementary Information

DeepPINK Results

To support the results obtained by SHAP, we also applied another method of determining feature importance called DeepPINK [28]. It works by comparing the original features with fake features. The knockoff features can be generated in many different ways, as long as they simulate the original data structure but are not related to the output. DeepPINK contrasts the relevance of the fake features against the regular input features to determine which ones are truly related to the output. It can also be used for feature selection with a controllable false discovery rate (FDR). It is worth highlighting the difficulty in feature selection in DNA methylation data. Most experiments have a couple dozen or a couple hundred samples. Depending on the type of platform used, the number of beta values for the CpG sites analyzed can vary from around 27 thousand to around 850 thousand. DeepPINK, even with a high FDR of 0.5, only selected 78 features. The fact that other sets of CpG sites unrelated to Horvath's 353 also perform similarly well emphasizes the difficulty in finding the "true" age-related CpG sites.

AltumAge captures relevant age-related CpG-CpG interactions The top 9 most important CpG sites according to SHAP account for 9.54% of total DeepPINK model importance.

Characterization of CpG sites by model interpretation. For the CTCF binding site analysis, the top 1000 CpG sites comprises 47.3% of DeepPINK importance. For the ChromHMM analysis, CpG importance values are also impacted by ChromHMM state for DeepPINK (Kruskal-Wallis H-test p-value = 2.982e-05). The chromatin state with the highest DeepPINK normalized median importance was heterochromatin (DeepPINK importance = 2.47e-14%, top 66th percentile of all CpG sites). Despite there being only 29 CpG sites characterized as heterochromatic, this result emphasizes the importance of chromatin packing with aging, as it is related to genome stability and maintenance.

Aging-related pathways. None of the CpG sites in SIRT genes appear very relevant for DeepPINK. cg21770145, located in SIRT7, accounts for 7.89e-12% of total DeepPINK importance and ranks 1342, with the highest SIRT DeepPINK importance value. For the mTOR pathway, cg05546044, located in MAPK1, has the highest DeepPINK importance of 0.029%, ranking 233. mTOR was not particularly relevant, with its most important CpG site being cg07029998 (DeepPINK importance = 1.12e-12%, rank 2459). All AMPK-related CpG sites had low (less than 10e-13%) DeepPINK importance values.

Figures and Tables

Supplementary Table 1: Evaluation metrics for all models in the validation set. The median absolute error (MAE) and the median error are in units of year, while the mean squared error (MSE) is in units of year-squared. R stands for Pearson's correlation coefficient. Bold numbers indicate better performance.

Model	CpGs	MAE	MSE	R	Median Error
AltumAge	20318	**3.337**	**59.712**	**0.953**	-0.922
AltumAge with ElasticNet CpGs	699	3.516	61.202	0.952	0.207
TabNet	20318	4.027	62.113	0.952	0.003
ElasticNet	699	3.708	67.981	0.947	-0.129
Random Forest	20318	6.206	131.126	0.895	**-0.001**
Support Vector Regression	20318	10.903	280.841	0.780	-2.668

Supplementary Table 2: List of ChromHMM states by ChromHMM state ID.

ChromHMM state ID	ChromHMM state
1	Active TSS
2	Flanking TSS
3	Flanking TSS Upstream
4	Flanking TSS Downstream
5	Strong transcription
6	Weak transcription
7	Genic enhancer1
8	Genic enhancer2
9	Active Enhancer 1
10	Active Enhancer 2
11	Weak Enhancer
12	ZNF genes and repeats
13	Heterochromatin
14	Bivalent/Poised TSS
15	Bivalent Enhancer
16	Repressed PolyComb
17	Weak Repressed PolyComb
18	Quiescent/Low

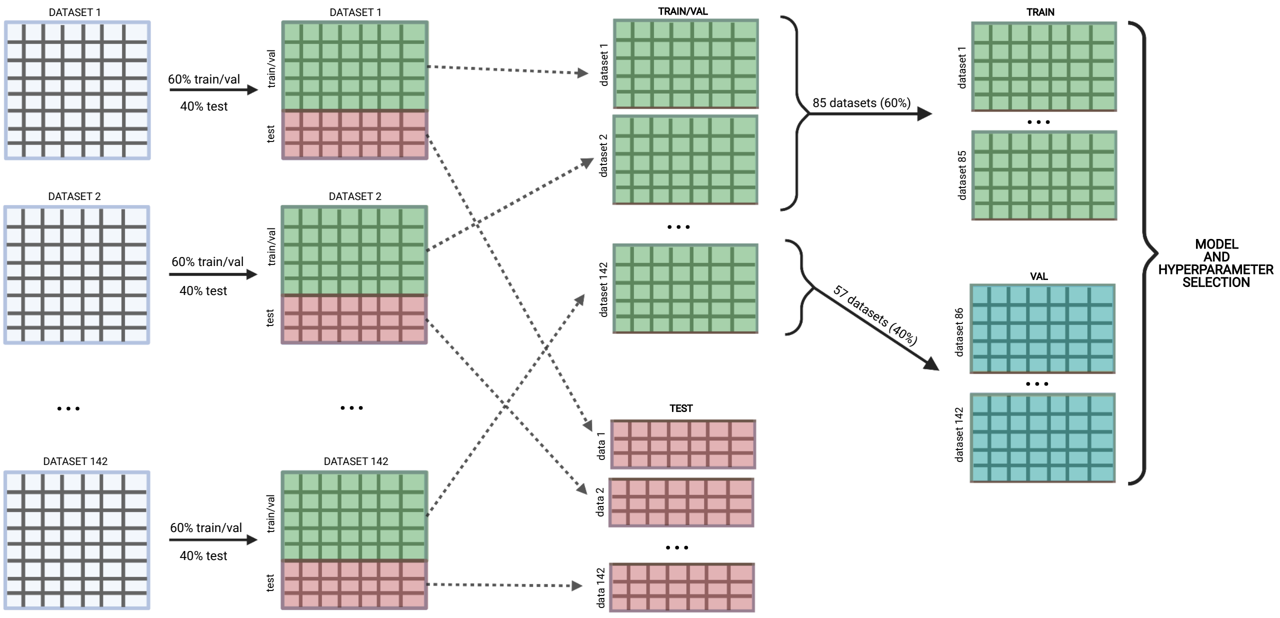

(a)

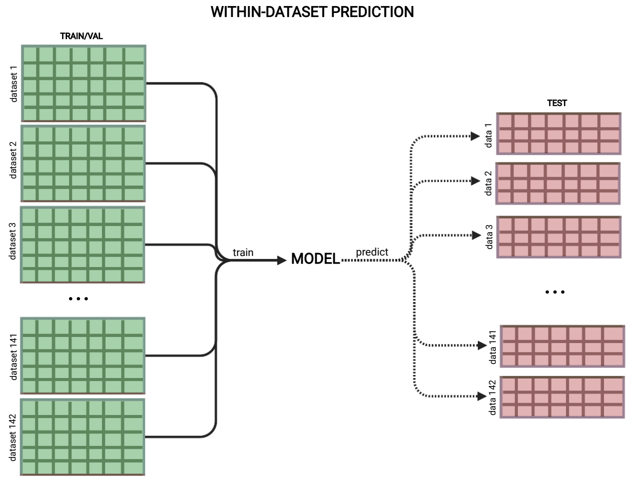

(b)

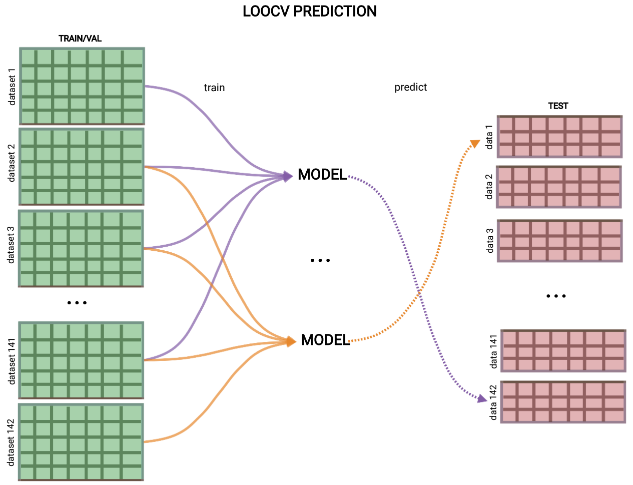

(c)

Supplementary Figure 1: Data split workflow. (a) Initially, each data set was randomly split 60% for training and validation and 40% for test. Then, the training data from 85 data sets (60% of training and validation) were used for training and the remaining 57 data sets (40% of training and validation) were used for validation. (b) For within-data set prediction, all training samples were used to train and predicted the entire test set. (c) For leave-one-data set-out cross validation (LOOCV) prediction, the training data from all data sets but one was used to train the model and predict the test set of the left-out data set. This process was repeated 142 times.

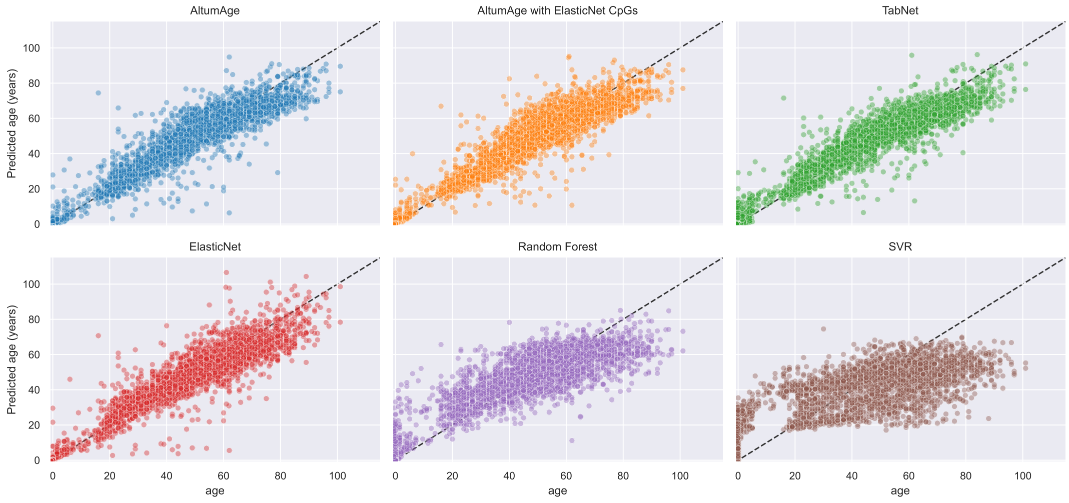

(a)

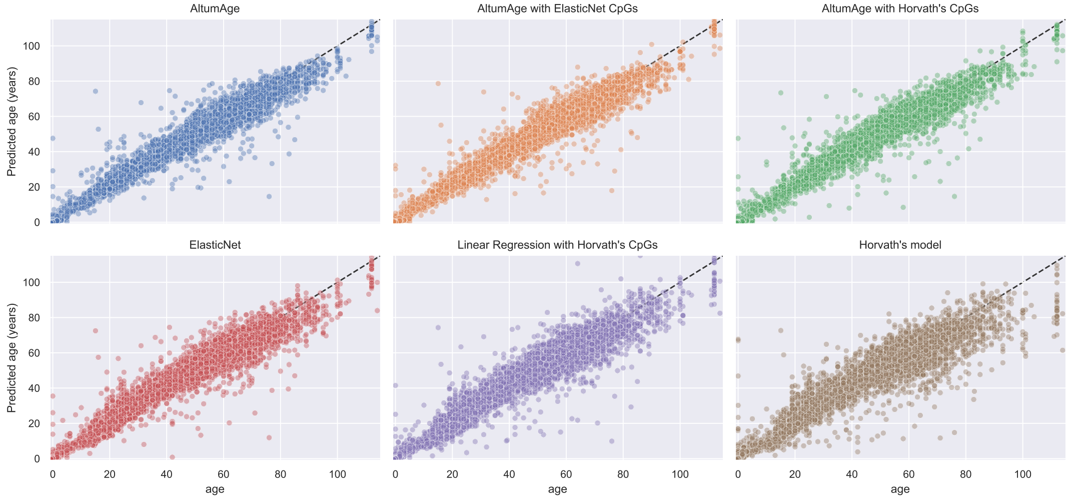

(b)

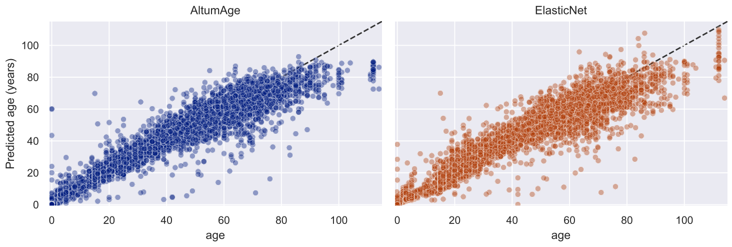

(c)

Supplementary Figure 2: Qualitative comparison of different models. (a) Models from model selection predicting the validation set. (b) Models from within-data set prediction in the test set. (c) Models from leave-one-data set-out cross validation (LOOCV) in the test set. In each panel, models are ordered from smallest to largest mean squared error (MSE). As shown by the closeness of the predictions to the true age line, AltumAge outperforms other models.

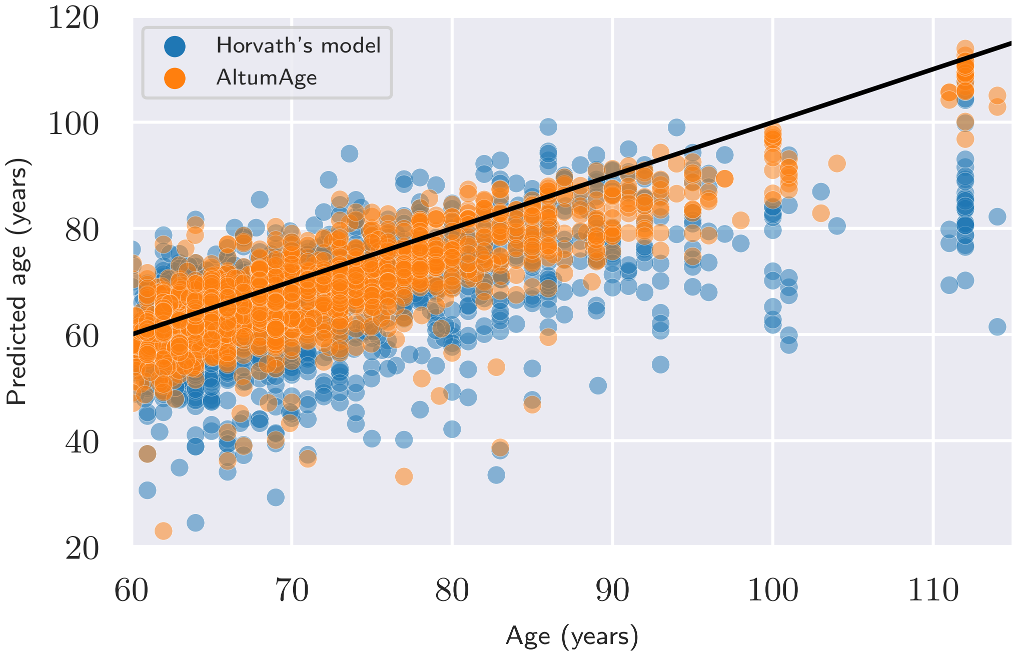

Supplementary Figure 3: Scatter plot showing the improved performance of AltumAge in comparison to Horvath's 2013 model for older ages. The black line represents the location where the predicted age equals the real age. AltumAge's predictions are generally closer to the black line. Horvath's predictions tends to give lower performance in higher ages.

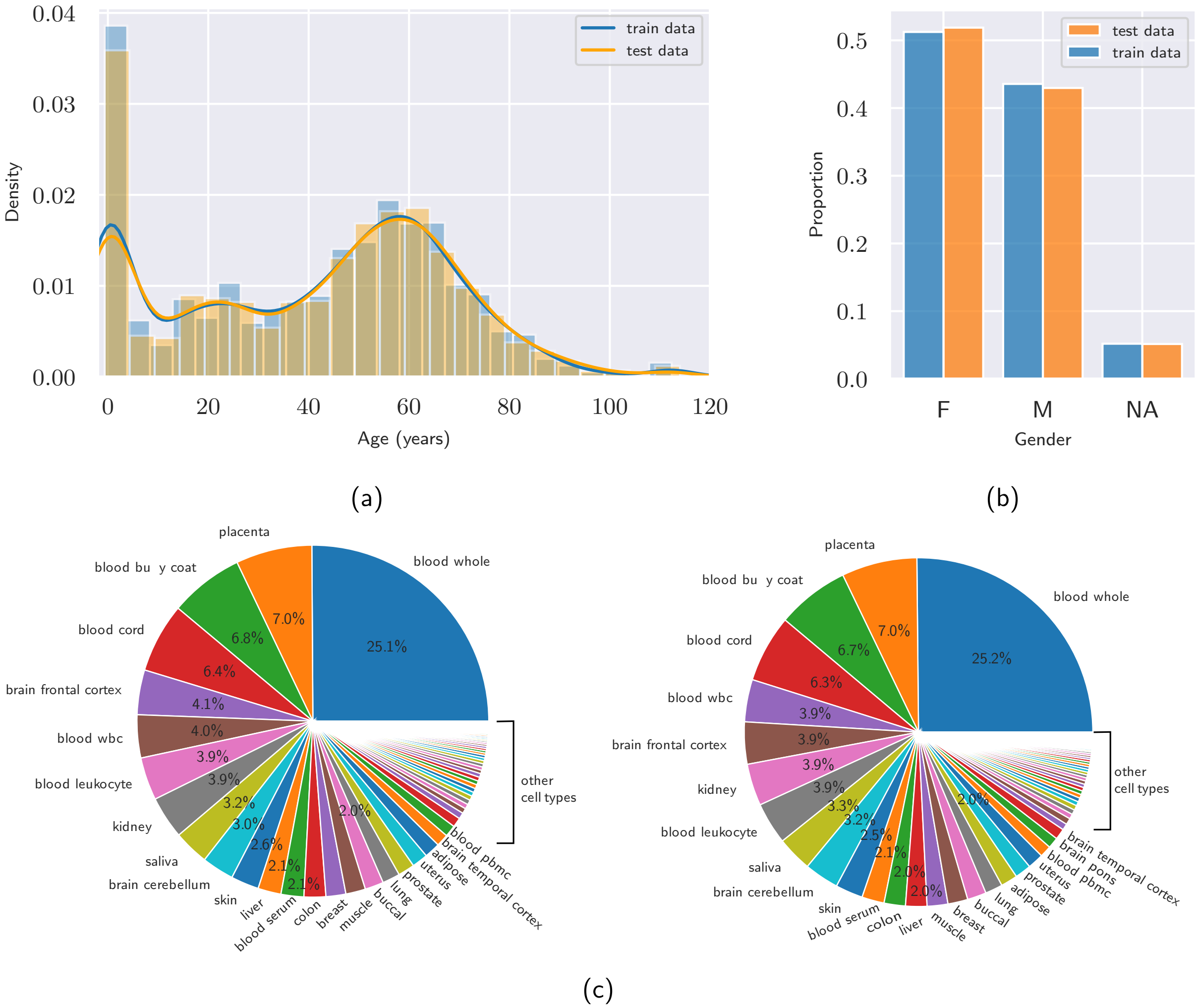

Supplementary Figure 4: Distribution of age, gender, and tissue type in both training and testing sets. The distributions of age (a) and gender (b) are virtually identical between the two sets. Similarly with the distribution of tissue type (c) in training (left) and testing (right).

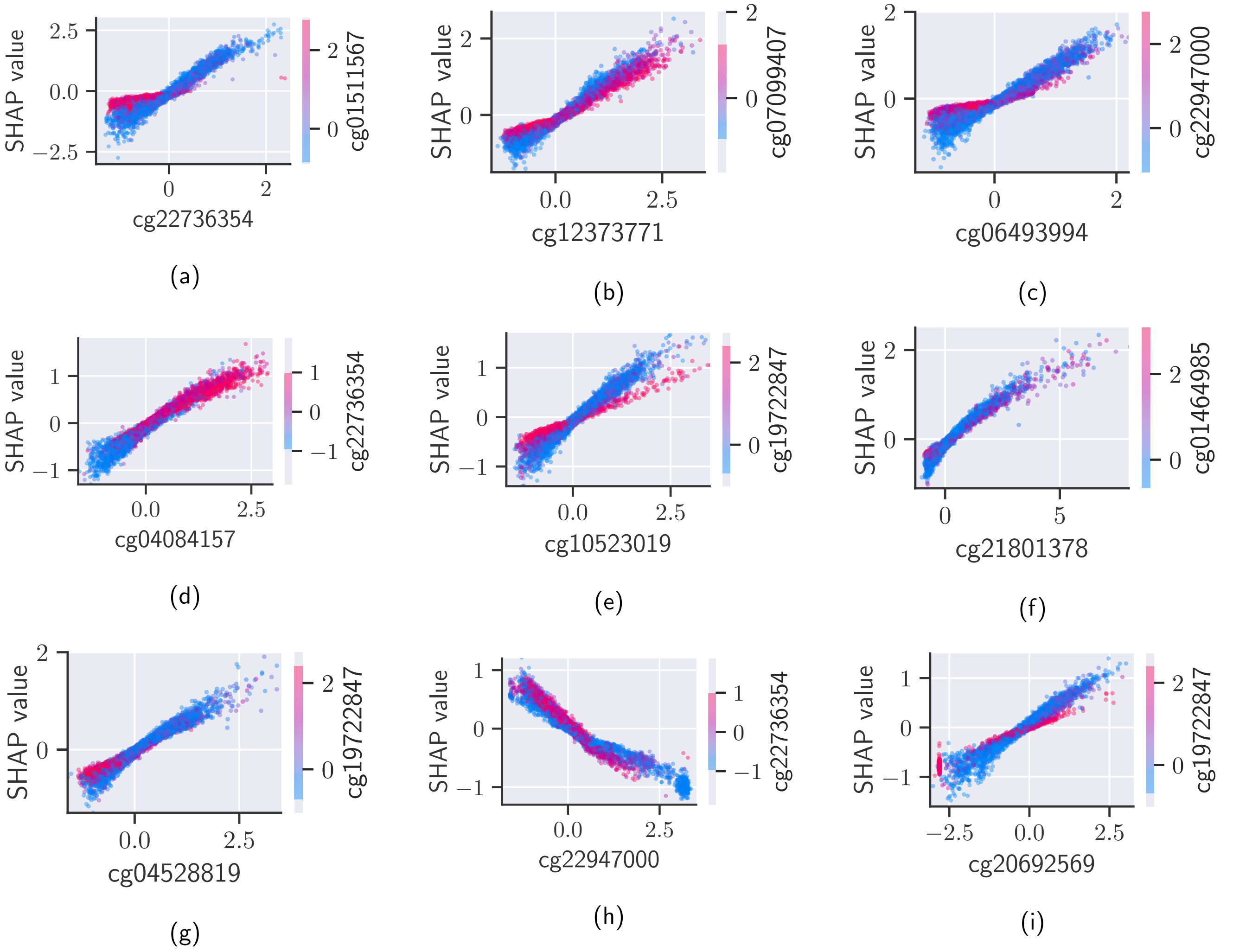

Supplementary Figure 5: Dependence plots of the nine most important CpG sites in AltumAge based on SHAP values. They are ordered from top left to bottom right (a-i) in terms of importance. The x-axis shows the scaled beta values for each specific CpG site; the y-axis, its SHAP value, and the coloring scheme, the scaled beta values for the CpG site with the highest interaction. The effect of a specific CpG site on the predicted age can vary based on a secondary CpG site.

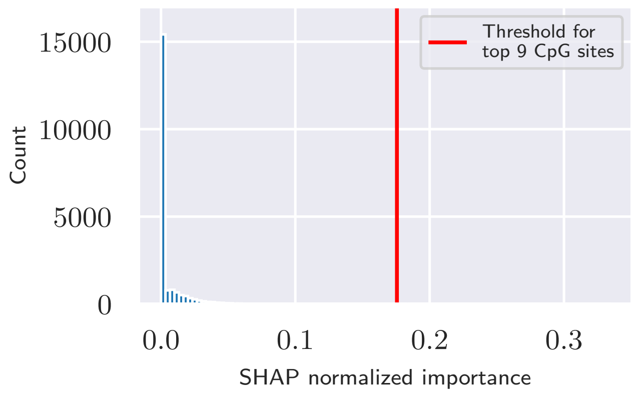

Supplementary Figure 6: Histogram of the normalized importance values of all AltumAge CpG sites according to SHAP. The red line represents the threshold for the top nine CpG sites. These have a much higher importance than most other CpG sites.

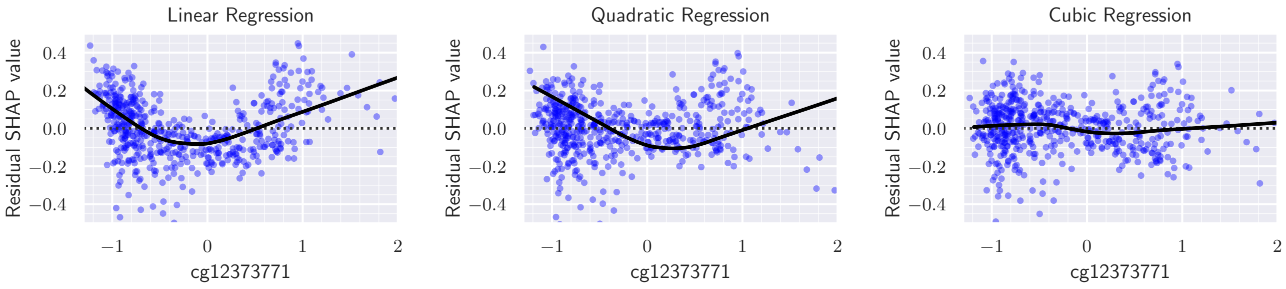

(a) (b) (c)

Supplementary Figure 7: Residual plots of cg12373771 by order of regression with lowess line. A linear regression (a) underestimates around the boundaries and underestimates in the middle, demonstrating the non-linear relationship. The same occurs with a quadratic regression (b). When the order is increased to three, the cubic regression (c) takes the non-linearity better into account.

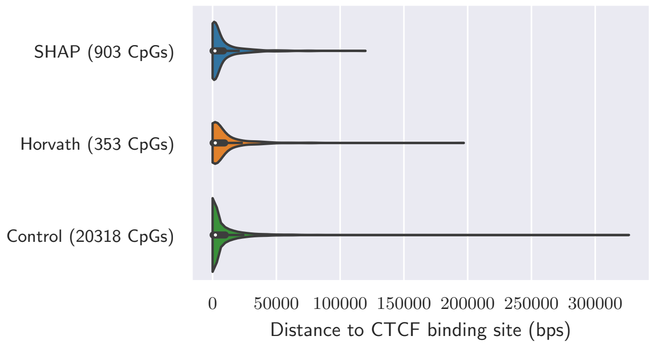

Supplementary Figure 8: Violin plot showing distance of CTCF binding sites for AltumAge's top CpGs based on SHAP and Horvath's CpGs. The top CpG sites according to SHAP are closer to CTCF binding sites than the 20,318 control CpG sites. Horvath's CpG sites are not statistically significantly different from the control.

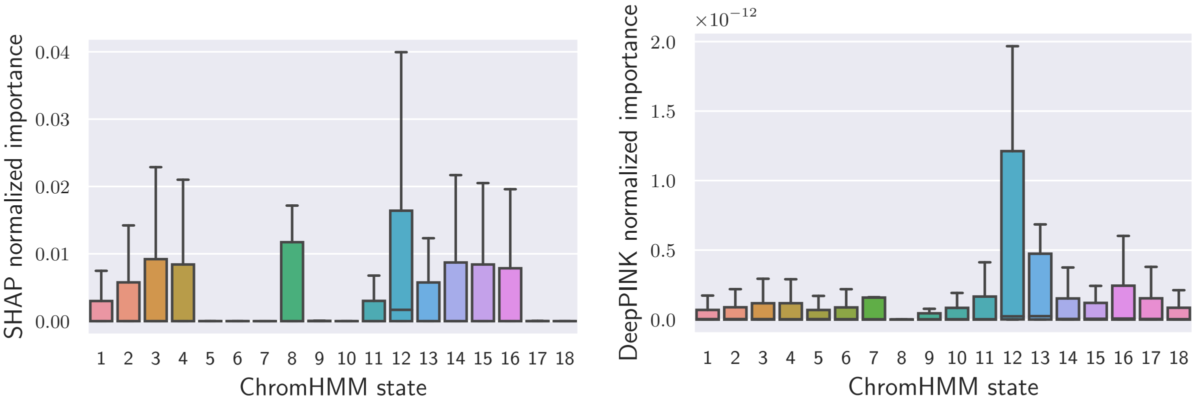

Supplementary Figure 9: Box plots of SHAP and DeepPINK normalized importance values by ChromHMM state. Outliers were removed for better figure visualization. No specific ChromHMM state stands out in importance. The box and whiskers show the quartiles of the data.

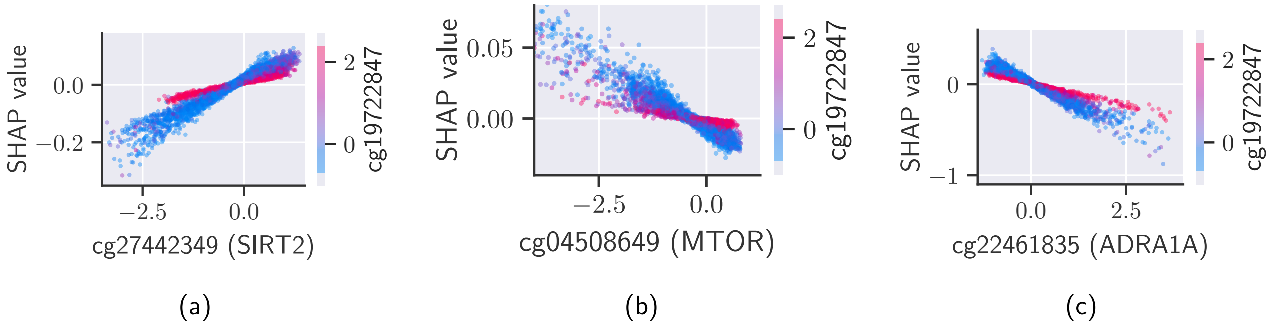

Supplementary Figure 10: SHAP dependence plots of three CpG sites in SIRT2, mTOR, and ADRA1A. The x-axis shows the standardized beta values for each specific CpG site; the y-axis, its SHAP value, and the coloring scheme, the scaled beta values for the CpG site with the highest interaction. These are the most important CpG sites according to SHAP for AltumAge in the SIRT and mTOR pathways.

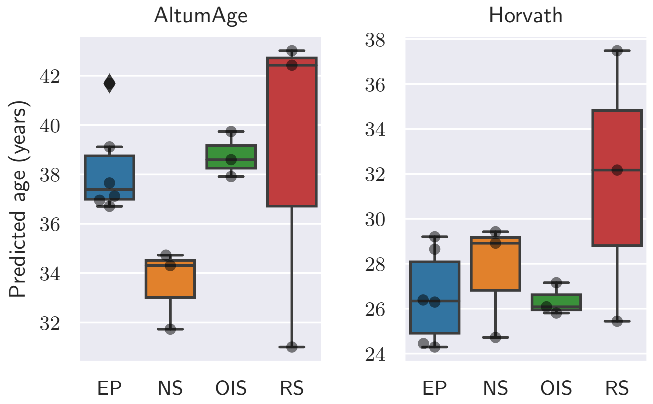

Supplementary Figure 11: Analysis of the effect of cellular senescence in data set GSE91069. Box plots showing predicted age for cultured fibroblasts in early passage (EP), near senescence (NS), oncogene-induced senescence (OIS), and replicative senescence (RS). The box and whiskers show the quartiles of the data.

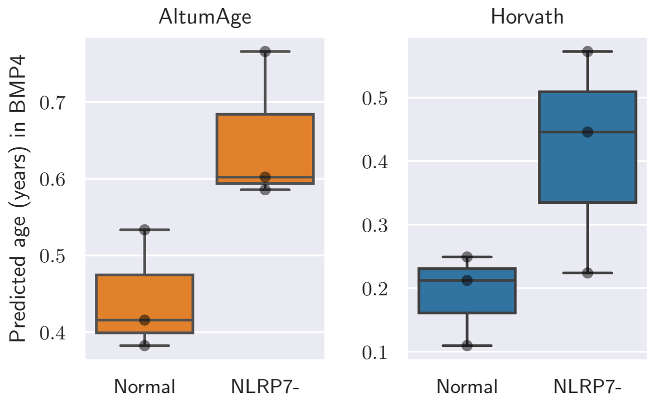

Supplementary Figure 12: Box plots showing predicted age of H9 ESCs with NLRP7 knockdown (NLRP7-) or control (Normal) in BMP4 differentiating medium. The box and whiskers show the quartiles of the data.
