# Supplementary material for: A pan-tissue DNA-methylation epigenetic clock based on deep learning
Source: NPJ Aging. 2022 Apr 19;8(1):4. doi: 10.1038/s41514-022-00085-y (PMC9158789; doi:10.1038/s41514-022-00085-y)
Supplement: Supplementary file 3 — Dataset details [file 41514_2022_85_MOESM3_ESM.pdf]

DATASET DESCRIPTION

TRAIN/VALIDATION/TEST

GSE27317: newborn umbilical cord buffy coat samples. All samples were recorded as having zero age. The platform used was Illumina's Infinium 27k Human Methylation Beadchip. (PMID: 21453505)

GSE41037: whole blood samples in schizophrenia patients and healthy subjects. Non-healthy patient samples were separated for further analysis and not included in training, validation, or testing. The platform used was Illumina's Infinium 27k Human Methylation Beadchip. (PMID: 23034122, 25424713, 26949191)

GSE38873: postmortem cerebellum samples from patients with schizophrenia or bipolar disorder or depression and healthy controls. Non-healthy patient samples were separated for further analysis and not included in training, validation, or testing. Out of 155 samples, there were 13 random replicates. Replicates were separated for further analysis and not included in training, validation, or testing. The platform used was Illumina's Infinium 27k Human Methylation Beadchip. (PMID: 25243493)

GSE15745: postmortem frozen brain samples from the cerebellum, frontal cortex, pons, and temporal cortex of each subject (four samples per subject). The platform used was Illumina's Infinium 27k Human Methylation Beadchip. (PMID: 20485568)

GSE32393: breast samples from women with or without breast cancer. Cancer samples were separated for further analysis and not included in training, validation, or testing. The platform used was Illumina's Infinium 27k Human Methylation Beadchip. (PMID: 22346766)

GSE25892: buccal epithelial cell samples from adolescents whose parents reported high levels of stress during the children's infancy. The platform used was Illumina's Infinium 27k Human Methylation Beadchip. (PMID: 21883162)

GSE20242: CD4+ T-cells and CD14+ monocytes samples. The platform used was Illumina's Infinium 27k Human Methylation Beadchip. (PMID: 20219945)

GSE22595: human dermal fibroblast samples expanded *in vitro*. The platform used was Illumina's Infinium 27k Human Methylation Beadchip. (PMID: 21347436)

GSE37988: liver samples from tumors or adjacent to tumors (two samples per patient). Cancer samples were separated for further analysis and not included in training, validation, or testing. The platform used was Illumina's Infinium 27k Human Methylation Beadchip. (PMID: 22976466)

GSE17448: mesenchymal stromal cells passaged *in vitro*. The platform used was Illumina's Infinium 27k Human Methylation Beadchip. (PMID: 19895632, 25241740)

GSE36642: blood mononuclear cell, human umbilical vascular endothelial cell, and placenta samples from monozygotic and dizygotic twins. Given that gestational week information was available, the age was encoded with the formula shown in Methods. The platform used was Illumina's Infinium 27k Human Methylation Beadchip. (PMID: 22800725)

GSE26126: prostate samples from normal and cancer tissue, and cultured prostate cells overexpressing DNMTs and EZH2. Cultured samples were disregarded. Cancer samples were separated for further analysis and not included in training, validation, or testing. The platform used was Illumina's Infinium 27k Human Methylation Beadchip. (PMID: 21521786)

GSE34035: saliva samples from alcohol users. The platform used was Illumina's Infinium 27k Human Methylation Beadchip. (PMID: 20386599)

GSE28746: saliva samples from pairs of identical twins. The platform used was Illumina's Infinium 27k Human Methylation Beadchip. (PMID: 21731603)

GSE20236: whole blood samples. The platform used was Illumina's Infinium 27k Human Methylation Beadchip. (PMID: 20219945)

GSE19711: whole blood samples from patients with or without ovarian cancer. Subjects with ovarian cancer were disregarded. The platform used was Illumina's Infinium 27k Human Methylation Beadchip. (PMID: 20219944)

GSE37008: peripheral blood mononuclear cell samples. Out of 94 samples, there were five random technical replicates. Replicates were discarded. The platform used was Illumina's Infinium 27k Human Methylation Beadchip. (PMID: 23045638)

GSE36812: blood cord samples from newborns. Given that gestational week information was available, the age was encoded with the formula shown in Methods. The platform used was Illumina's Infinium 27k Human Methylation Beadchip.

GSE34257: blood cord samples and whole blood samples from 9-month-old infants. Blood cord samples were recorded as having zero age. The platform used was Illumina's Infinium 27k Human Methylation Beadchip. (PMID: 22307237)

GSE38608: postmortem brain cerebellum and occipital cortex samples from subjects with autism and healthy controls. Non-healthy patient samples were separated for further analysis and not included in training, validation, or testing. Out of 34 samples, there were three replicates. Replicates were separated for further analysis and not included in training, validation, or testing. The platform used was Illumina's Infinium 27k Human Methylation Beadchip. (PMID: 22984548)

GSE38291: muscle and adipose samples from monozygotic twins discordant for type 2 diabetes. Non-healthy patient samples were separated for further analysis and not included in training, validation, or testing. The platform used was Illumina's Infinium 27k Human Methylation Beadchip. (PMID: 23251491)

GSE36166: muscle samples from subjects receiving a control or a high-fat diet. Both groups were included. The platform used was Illumina's Infinium 27k Human Methylation Beadchip. (PMID: 22961225)

GSE63384: lung samples from tumors or adjacent to tumors (two samples per patient). Cancer samples were separated for further analysis and not included in training, validation, or testing. The platform used was Illumina's Infinium 27k Human Methylation Beadchip. (PMID: 26134223)

GSE59274: placental tissue samples from women with uncomplicated pregnancies and with preeclampsia. Both groups were included. Given that gestational week information was available, the age was encoded with the formula shown in Methods. The platform used was Illumina's Infinium 27k Human Methylation Beadchip. (PMID: 25247495)

GSE57285: whole blood samples from women with BRCA1 wild-type or mutants, and with or without breast cancer. Subjects with cancer were disregarded. BRCA1 mutant patient samples were separated for further analysis and not included in training, validation, or testing. The platform used was Illumina's Infinium 27k Human Methylation Beadchip. (PMID: 25067956)

GSE56606: CD14+ monocyte samples from monozygotic twins discordant for type 1 diabetes. Non-healthy patient samples were separated for further analysis and not included in training, validation, or testing. The platform used was Illumina's Infinium 27k Human Methylation Beadchip. (PMID: 21980303)

GSE49908: skeletal muscle samples. The platform used was Illumina's Infinium 27k Human Methylation Beadchip. (PMID: 24034465)

GSE49907: kidney samples. The platform used was Illumina's Infinium 27k Human Methylation Beadchip. (PMID: 24034465)

GSE49905: brain cerebral cortex samples. The platform used was Illumina's Infinium 27k Human Methylation Beadchip. (PMID: 24034465)

GSE49904: blood buffy coat samples. The platform used was Illumina's Infinium 27k Human Methylation Beadchip. (PMID: 24034465)

E-GEOD-43256: placental chorionic villi from electively terminated pregnancies or miscarriages. Both groups were included. Given that gestational week information was available, the age was encoded with the formula shown in Methods. The platform used was Illumina's Infinium 27k Human Methylation Beadchip. (PMID: 23583422)

E-GEOD-64940: cord blood samples from newborns. All samples were encoded as zero age. The platform used was Illumina's Infinium 27k Human Methylation Beadchip. (PMID: 25742137)

E-MTAB-2344: white blood cell samples in patients with stroke and/or obesity and healthy controls. Non-healthy patient samples were separated for further analysis and not included in training, validation, or testing. The platform used was Illumina's Infinium 27k Human Methylation Beadchip. (PMID: 25429063)

E-GEOD-62867: advanced atherosclerotic plaques, atherosclerotic-resistant internal mammary arteries, and great saphenous veins samples. Non-healthy patient samples were separated for further analysis and not included in training, validation, or testing. The platform used was Illumina's Infinium 27k Human Methylation Beadchip. (PMID: 25856389)

E-GEOD-44763: whole blood samples from obese and lean subjects. Non-healthy patient samples were separated for further analysis and not included in training, validation, or testing. The platform used was Illumina's Infinium 27k Human Methylation Beadchip. (PMID: 25010727)

E-GEOD-48988: colon samples from subjects with different lifestyle factors. The platform used was Illumina's Infinium 27k Human Methylation Beadchip. (PMID: 24973978)

E-GEOD-58119: serum samples from healthy postmenopausal women. The platform used was Illumina's Infinium 27k Human Methylation Beadchip. (PMID: 25067956)

E-GEOD-58045: whole blood samples. The platform used was Illumina's Infinium 27k Human Methylation Beadchip. (PMID: 22532803)

E-GEOD-57484: whole blood samples from normal and obese children. Non-healthy patient samples were separated for further analysis and not included in training, validation, or testing. The platform used was Illumina's Infinium 27k Human Methylation Beadchip.

E-GEOD-54211: buccal samples from oral lichenoid disease patients and healthy controls. Non-healthy patient samples were not included in training, validation, or testing. The platform used was Illumina's Infinium 27k Human Methylation Beadchip.

E-GEOD-56342: small airway bronchioles samples from chronic obstructive pulmonary disease and healthy controls. Non-healthy patient samples were separated for further analysis and not included in training, validation, or testing. The platform used was Illumina's Infinium 27k Human Methylation Beadchip. (PMID: 24298892)

E-GEOD-27044: peripheral blood leukocytes from autistic children and unaffected siblings. Non-healthy patient samples were separated for further analysis and not included in training, validation, or testing. The platform used was Illumina's Infinium 27k Human Methylation Beadchip. (PMID: 22300631)

E-GEOD-36194: postmortem cerebellum and frontal cortex samples (two samples per subject). The platform used was Illumina's Infinium 27k Human Methylation Beadchip.

E-GEOD-21232: pancreatic islet samples from patients with type 2 diabetes and healthy controls. Non-healthy patient samples were separated for further analysis and not included in training, validation, or testing. The platform used was Illumina's Infinium 27k Human Methylation Beadchip. (PMID: 22293752)

E-GEOD-32867: lung tumor and adjacent non-tumor lung samples. Cancer samples were separated for further analysis and not included in training, validation, or testing. The platform used was Illumina's Infinium 27k Human Methylation Beadchip. (PMID: 22613842)

E-GEOD-30759: uterine cervix and cervical cancer samples. Cancer samples were separated for further analysis and not included in training, validation, or testing. The platform used was Illumina's Infinium 27k Human Methylation Beadchip. (PMID: 22346766, 22453031, 27103033)

E-GEOD-30758: uterine cervix samples. The platform used was Illumina's Infinium 27k Human Methylation Beadchip. (PMID: 22346766, 22453031, 27103033)

E-GEOD-32396: whole blood sample from women with or without BRCA1 mutation, with or without breast cancer. Cancer samples were separated for further analysis and not included in training, validation, or testing. The platform used was Illumina's Infinium 27k Human Methylation Beadchip. (PMID: 22346766)

E-GEOD-31979: breast tumor and adjacent non-tumor breast samples. Cancer samples were separated for further analysis and not included in training, validation, or testing. The platform used was Illumina's Infinium 27k Human Methylation Beadchip. (PMID: 21825015)

E-MTAB-625: epidermis samples. The platform used was Illumina's Infinium 27k Human Methylation Beadchip. (PMID: 21993563)

E-MTAB-487: CD34+ hematopoietic progenitor cell, CD16+ granulocytes, CD14+ monocytes, mesenchymal stem cells, keratinocytes, and fibroblast samples. The platform used was Illumina's Infinium 27k Human Methylation Beadchip. (PMID: 21427290)

GSE90124: skin peri-umbilical punch samples. The platform used was Illumina's Infinium 450k Human Methylation Beadchip. (PMID: 27993549)

GSE115797: skin samples from psoriatic skin disease and adjacent normal skin samples. Non-healthy patient samples were separated for further analysis and not included in training, validation, or testing. The platform used was Illumina's Infinium 450k Human Methylation Beadchip. (PMID: 30092825)

GSE99624: whole blood samples from osteoporotic and healthy control patients. Non-healthy patient samples were separated for further analysis and not included in training, validation, or testing. The platform used was Illumina's Infinium 450k Human Methylation Beadchip. (PMID: 28926142)

GSE108213: breast samples. The platform used was Illumina's Infinium 450k Human Methylation Beadchip. (PMID: 30821641)

GSE92767: saliva samples. The platform used was Illumina's Infinium 450k Human Methylation Beadchip. (PMID: 28419903)

GSE69176: umbilical cord blood samples from newborns. All ages were encoded as zero age. The platform used was Illumina's Infinium 450k Human Methylation Beadchip.

GSE40360: postmortem frontal lobe white matter in multiple sclerosis patients and healthy controls. Non-healthy patient samples were separated for further analysis and not included in training, validation, or testing. The platform used was Illumina's Infinium 450k Human Methylation Beadchip. (PMID: 24270187)

GSE59157: kidney samples from tumor patients and healthy subjects. Cancer samples were separated for further analysis and not included in training, validation, or testing. The platform used was Illumina's Infinium 450k Human Methylation Beadchip. (PMID: 25134821)

GSE42861: peripheral blood leukocytes from rheumatoid arthritis patients and healthy controls. Samples from rheumatoid arthritis patients were discarded. The platform used was Illumina's Infinium 450k Human Methylation Beadchip. (PMID: 29921915)

E-MTAB-2372: white blood cell samples from obese patients subject to two different diets. Both groups were included. The platform used was Illumina's Infinium 450k Human Methylation Beadchip.

E-GEOD-73832: intestinal samples from patients with small intestinal neuroendocrine tumors and healthy controls. Samples from patients with cancer were not included in training, validation, or testing. The platform used was Illumina's Infinium 450k Human Methylation Beadchip. (PMID: 26169971, 28049633)

E-GEOD-71678: placental tissue samples exposed to different levels of arsenic. All groups were included. All ages were encoded as zero age. The platform used was Illumina's Infinium 450k Human Methylation Beadchip. (PMID: 26771251, 29373860)

E-GEOD-71245: blood samples from different types of blood cells. The platform used was Illumina's Infinium 450k Human Methylation Beadchip. (PMID: 26291385)

E-GEOD-83334: whole blood and cord blood from newborns and infants measured longitudinally. The platform used was Illumina's Infinium 450k Human Methylation Beadchip. (PMID: 27259700)

E-GEOD-75248: placental samples from newborns. All ages were encoded as zero age. The platform used was Illumina's Infinium 450k Human Methylation Beadchip. (PMID: 27366929, 29373860, 28621618)

E-GEOD-77955: liver, colon, and small intestine samples from patients with and without cancer. Cancer samples were separated for further analysis and not included in training, validation, or testing. The platform used was Illumina's Infinium 450k Human Methylation Beadchip. (PMID: 27270421)

E-GEOD-67705: blood samples from HIV+ and HIV- subjects. Non-healthy patient samples were separated for further analysis and not included in training, validation, or testing. The platform used was Illumina's Infinium 450k Human Methylation Beadchip. (PMID: 27105112)

E-GEOD-77445: whole blood samples from subjects with different stress levels. All groups were included. The platform used was Illumina's Infinium 450k Human Methylation Beadchip. (PMID: 26997371)

E-GEOD-79056: cord blood samples. Given that gestational week information was available, the age was encoded with the formula shown in Methods. The platform used was Illumina's Infinium 450k Human Methylation Beadchip.

E-GEOD-72556: saliva samples from children. The platform used was Illumina's Infinium 450k Human Methylation Beadchip.

E-GEOD-52068: nasopharyngeal epithelial tissue from tumors or healthy controls. Cancer samples were separated for further analysis and not included in training, validation, or testing. The platform used was Illumina's Infinium 450k Human Methylation Beadchip. (PMID: 26443805, 28146149)

E-GEOD-74738: placental chorionic villi samples with control pregnancies and several disorders. Non-healthy patient samples were separated for further analysis and not included in training, validation, or testing. Samples from maternal tissue were excluded. The platform used was Illumina's Infinium 450k Human Methylation Beadchip. (PMID: 26769960)

E-GEOD-76105: postmortem brain superior temporal gyrus of Alzheimer's disease and control subjects. Non-healthy patient samples were not included in training, validation, or testing. The platform used was Illumina's Infinium 450k Human Methylation Beadchip. (PMID: 26769960)

E-GEOD-65638: whole blood samples twins. The platform used was Illumina's Infinium 450k Human Methylation Beadchip. (PMID: 26635134)

E-GEOD-71955: CD4+ and CD8+ T-cell samples from subjects with Graves' disease or healthy controls. Non-healthy patient samples were separated for further analysis and not included in training, validation, or testing. The platform used was Illumina's Infinium 450k Human Methylation Beadchip. (PMID: 26459776)

E-GEOD-63106: knee and hip cartilage from patients who underwent joint replacement surgery primarily due to osteoarthritis. The platform used was Illumina's Infinium 450k Human Methylation Beadchip.

E-GEOD-73377: placental tissue samples from healthy and preeclamptic women. Both groups were included. Given that gestational week information was available, the age was encoded with the formula shown in Methods. The platform used was Illumina's Infinium 450k Human Methylation Beadchip. (PMID: 26510177)

E-GEOD-56515: muscle, amnion, pancreas, and adrenal gland fetal tissues. Given that gestational week information was available, the age was encoded with the formula shown in Methods. The platform used was Illumina's Infinium 450k Human Methylation Beadchip. (PMID: 26492326)

E-GEOD-73103: whole blood samples. The platform used was Illumina's Infinium 450k Human Methylation Beadchip. (PMID: 26449484)

E-GEOD-67024: abdominal subcutaneous fat cells from obese and healthy subjects. Non-healthy patient samples were separated for further analysis and not included in training, validation, or testing. The platform used was Illumina's Infinium 450k Human Methylation Beadchip. (PMID: 26351548)

E-GEOD-72338: neutrophils and monocytes from patients with tuberculosis and household controls. Non-healthy patient samples were separated for further analysis and not included in training, validation, or testing. The platform used was Illumina's Infinium 450k Human Methylation Beadchip. (PMID: 26351548)

E-GEOD-59457: various postmortem brain region tissue samples from HIV+ and HIV- subjects. Non-healthy patient samples were separated for further analysis and not included in training, validation, or testing. The platform used was Illumina's Infinium 450k Human Methylation Beadchip. (PMID: 25969563)

E-GEOD-64511: human bone, brain tissue samples, and 30 tissues from a 112-year-old woman. The platform used was Illumina's Infinium 450k Human Methylation Beadchip. (PMID: 26000617)

E-GEOD-64495: whole blood from subjects with or without developmental disorder syndrome X. Non-healthy patient samples were separated for further analysis and not included in training, validation, or testing. The platform used was Illumina's Infinium 450k Human Methylation Beadchip. (PMID: 25991677)

E-GEOD-59509: whole blood, saliva, menstrual blood, vaginal swab, and semen samples. Semen samples were not included. The platform used was Illumina's Infinium 450k Human Methylation Beadchip. (PMID: 25796047)

E-GEOD-67444: neonatal blood samples. All ages were encoded as zero age. The platform used was Illumina's Infinium 450k Human Methylation Beadchip.

E-GEOD-62219: longitudinal peripheral blood leukocyte samples from infants. The platform used was Illumina's Infinium 450k Human Methylation Beadchip. (PMID: 25874017)

E-GEOD-51954: epidermal and dermal samples from sun-exposed and sun-protected body sites. Sun-exposed patient samples were separated for further analysis and not included in training, validation, or testing. The platform used was Illumina's Infinium 450k Human Methylation Beadchip. (PMID: 25886480)

E-GEOD-52588: blood samples from subjects with or without Down syndrome. Non-healthy patient samples were separated for further analysis and not included in training, validation, or testing. The platform used was Illumina's Infinium 450k Human Methylation Beadchip. (PMID: 25701644, 25678027, 25701668)

E-GEOD-36054: peripheral blood leukocytes from children. The platform used was Illumina's Infinium 450k Human Methylation Beadchip. (PMID: 22300631)

E-GEOD-50660: peripheral blood from never, former, and current smokers. Current smokers' samples were separated for further analysis and not included in training, validation, or testing. The platform used was Illumina's Infinium 450k Human Methylation Beadchip. (PMID: 25424692)

E-GEOD-61259: muscle samples. The platform used was Illumina's Infinium 450k Human Methylation Beadchip. (PMID: 25313081)

E-GEOD-61258: liver samples. The platform used was Illumina's Infinium 450k Human Methylation Beadchip. (PMID: 25313081)

E-GEOD-61257: adipose samples. The platform used was Illumina's Infinium 450k Human Methylation Beadchip. (PMID: 25313081)

E-GEOD-61454: liver, muscle, and subcutaneous adipose tissue samples. The platform used was Illumina's Infinium 450k Human Methylation Beadchip. (PMID: 25282492)

E-GEOD-61380: postmortem prefrontal cortex brain samples from subjects with schizophrenia and controls. Non-healthy patient samples were separated for further analysis and not included in training, validation, or testing. The platform used was Illumina's Infinium 450k Human Methylation Beadchip. (PMID: 26351548)

E-GEOD-54690: blood samples from subjects with or without dietary flavanol supplementation. Both groups were included. The platform used was Illumina's Infinium 450k Human Methylation Beadchip. (PMID: 24763279)

E-GEOD-49149: pancreatic ductal adenocarcinoma samples and normal adjacent tissues. Cancer samples were separated for further analysis and not included in training, validation, or testing. The platform used was Illumina's Infinium 450k Human Methylation Beadchip. (PMID: 24500968, 26909576)

E-GEOD-55438: placental samples from the first and third trimesters. They were encoded as being one month and half and seven months and a half into the pregnancy, respectively; the age was encoded with

the formula shown in Methods. The platform used was Illumina’s Infinium 450k Human Methylation Beadchip. (PMID: 24951174)

E-GEOD-61107: postmortem brain samples from subjects with schizophrenia and controls. Non-healthy patient samples were separated for further analysis and not included in training, validation, or testing. The platform used was Illumina’s Infinium 450k Human Methylation Beadchip. (PMID: 24399042)

E-GEOD-53740: blood samples from progressive supranuclear palsy patients, frontotemporal dementia patients, and healthy controls. Non-healthy patient samples were separated for further analysis and not included in training, validation, or testing. The platform used was Illumina’s Infinium 450k Human Methylation Beadchip. (PMID: 24603599, 25589773)

E-GEOD-57767: placental tissue samples from term and preeclampsia pregnancies. Only normal pregnancies were included with zero age, as the other samples did not have gestational week. The platform used was Illumina’s Infinium 450k Human Methylation Beadchip. (PMID: 24963923)

E-GEOD-49064: peripheral blood mononucleated cells cultured in vitro with or without the synthetic transcription modulating agent WY14,643. Samples cultured with WY14,643 were removed. The platform used was Illumina’s Infinium 450k Human Methylation Beadchip. (PMID: 24789080)

E-GEOD-50759: ectodermal cells of autism disorder patients and healthy controls. Non-healthy patient samples were separated for further analysis and not included in training, validation, or testing. The platform used was Illumina’s Infinium 450k Human Methylation Beadchip. (PMID: 24875834)

E-GEOD-56553: peripheral mononuclear blood cells of asthmatic patients. The platform used was Illumina’s Infinium 450k Human Methylation Beadchip. (PMID: 24963923)

E-GEOD-54446: umbilical cord samples. All ages were encoded as zero age. The platform used was Illumina’s Infinium 450k Human Methylation Beadchip. (PMID: 24709820)

E-GEOD-54399: cord blood and placenta. Samples C1 and C20 were contaminated and were not included. All ages were encoded as zero age. The platform used was Illumina’s Infinium 450k Human Methylation Beadchip. (PMID: 29017633, 25043696, 28680507)

E-GEOD-53162: postmortem brain samples from autism cases and controls. Non-healthy patient samples were separated for further analysis and not included in training, validation, or testing. The platform used was Illumina’s Infinium 450k Human Methylation Beadchip. (PMID: 23999529)

E-GEOD-53128: whole blood samples. The platform used was Illumina’s Infinium 450k Human Methylation Beadchip. (PMID: 24496475)

E-GEOD-50498: muscle samples. The platform used was Illumina’s Infinium 450k Human Methylation Beadchip. (PMID: 24304487)

E-GEOD-47513: subcutaneous abdominal and gluteal adipose tissue samples. The platform used was Illumina’s Infinium 450k Human Methylation Beadchip. (PMID: 24340035)

E-GEOD-49393: postmortem prefrontal cortex tissues of individuals with alcohol use disorders or controls. Non-healthy patient samples were separated for further analysis and not included in training, validation, or testing. The platform used was Illumina's Infinium 450k Human Methylation Beadchip. (PMID: 24163133, 26763658)

E-GEOD-39004: breast tissue samples with or without tumors. Cancer samples were separated for further analysis and not included in training, validation, or testing. The platform used was Illumina's Infinium 450k Human Methylation Beadchip. (PMID: 24316975, 30501643, 25016594)

E-GEOD-51388: blood samples taken longitudinally from monozygotic twins. The platform used was Illumina's Infinium 450k Human Methylation Beadchip. (PMID: 26248206)

E-GEOD-51032: blood buffy coat. The platform used was Illumina's Infinium 450k Human Methylation Beadchip.

E-GEOD-48325: liver samples from patients with non-alcoholic fatty liver disease and healthy controls. Non-healthy patient samples were separated for further analysis and not included in training, validation, or testing. The platform used was Illumina's Infinium 450k Human Methylation Beadchip. (PMID: 23931760, 25313081)

E-GEOD-44712: placental tissue from normal and preeclamptic pregnancies. Both groups were included. Given that gestational week information was available, the age was encoded with the formula shown in Methods. The platform used was Illumina's Infinium 450k Human Methylation Beadchip. (PMID: 23770704)

E-GEOD-45461: B cell precursor cells from 20-24 weeks of gestation. Age was encoded as 22 gestational weeks and transformed with the formula shown in Methods. The platform used was Illumina's Infinium 450k Human Methylation Beadchip. (PMID: 23074194)

E-GEOD-40279: whole blood samples. The platform used was Illumina's Infinium 450k Human Methylation Beadchip. (PMID: 23177740)

E-GEOD-41169: whole blood of schizophrenia and healthy subjects. Non-healthy patient samples were separated for further analysis and not included in training, validation, or testing. The platform used was Illumina's Infinium 450k Human Methylation Beadchip. (PMID: 25969563)

E-GEOD-32149: peripheral blood from ulcerative colitis, Crohn's disease, and healthy subjects. Non-healthy patient samples were separated for further analysis and not included in training, validation, or testing. The platform used was Illumina's Infinium 450k Human Methylation Beadchip.

E-GEOD-41826: brain frontal cortex samples from depression and healthy subjects. Non-healthy patient samples were separated for further analysis and not included in training, validation, or testing. The platform used was Illumina's Infinium 450k Human Methylation Beadchip. (PMID: 23426267)

E-GEOD-42700: buccal samples from monozygotic and dizygotic twins at birth and at 18 months of age. The platform used was Illumina's Infinium 450k Human Methylation Beadchip. (PMID: 23697701)

E-GEOD-32146: normal, Crohn's disease, and ulcerative colitis colon mucosa. Non-healthy patient samples were separated for further analysis and not included in training, validation, or testing. The platform used was Illumina's Infinium 450k Human Methylation Beadchip.

E-GEOD-30870: cord blood and whole blood from newborns and nonagenarians. Age from newborns was encoded as zero age. The platform used was Illumina's Infinium 450k Human Methylation Beadchip. (PMID: 26161907)

E-GEOD-34639: CD4+ T-cells from subjects with or without dietary allergies. Both groups were included. The platform used was Illumina's Infinium 450k Human Methylation Beadchip. (PMID: 22495533, 24762976)

E-GEOD-63347: various brain samples from patients with or without autism. Non-healthy patient samples were separated for further analysis and not included in training, validation, or testing. The platform used was Illumina's Infinium 450k Human Methylation Beadchip. (PMID: 25678027)

E-GEOD-59592: blood from infants exposed to varying degrees of aflatoxin B1. All groups were included. The platform used was Illumina's Infinium 450k Human Methylation Beadchip. (PMID: 25855716, 26062908, 29034560)

GSE77241: astrocytoma, fetal brain, and adult brain tissue samples. Cancer samples were disregarded. The platform used was Illumina's Infinium 450k Human Methylation Beadchip. (PMID: 27229157)

GSE148000: sputum samples from subjects with asthma, COPD, and healthy controls. Non-healthy patient samples were separated for further analysis and not included in training, validation, or testing. The platform used was Illumina's Infinium 450k Human Methylation Beadchip. (PMID: 33076907)

TCGA-LUSC: bronchus and lung, normal and cancer. Cancer samples were not included in training, validation, or testing.

TCGA-THCA: thyroid gland, normal and cancer. Cancer samples were not included in training, validation, or testing.

TCGA-HNSC: head and neck, normal and cancer. Cancer samples were not included in training, validation, or testing.

TCGA-KIRC: kidney, normal and cancer. Cancer samples were not included in training, validation, or testing.

TCGA-KIRP: kidney, normal and cancer. Cancer samples were not included in training, validation, or testing.

TCGA-LUAD: bronchus and lung, normal and cancer. Cancer samples were not included in training, validation, or testing.

TCGA-PRAD: prostate, normal and cancer. Cancer samples were not included in training, validation, or testing.

TCGA-STAD: stomach, normal and cancer. Cancer samples were not included in training, validation, or testing.

TCGA-COAD: colon, normal and cancer. Cancer samples were not included in training, validation, or testing.

TCGA-LIHC: liver and intrahepatic bile ducts, normal and cancer. Cancer samples were not included in training, validation, or testing.

TCGA-UCEC: uterus, normal and cancer. Cancer samples were not included in training, validation, or testing.

TCGA-BRCA: breast, normal and cancer. Cancer samples were not included in training, validation, or testing.

RELIABILITY

GSE55763: whole blood from 2,664 human samples and 36 technical replicates. Only the 36 duplicates were included for the analysis of reliability. The platform used was Illumina's Infinium 450k Human Methylation Beadchip. (PMID: 25853392, 28002404)

IN VITRO

GSE91069: 31 samples with different groups of transformation and senescent cells. Fibroblasts were subdivided in early passage, near senescence, oncogene-induced senescence, and replicative senescence. Only these four groups were used for analysis. The platform used was Illumina's Infinium 450k Human Methylation Beadchip. (PMID: 29438699)

GSE30653: hESCs and hiPSC samples representing over 100 cell lines. The platform used was Illumina's Infinium 27k Human Methylation Beadchip. (PMID: 22560082)

GSE100249: HEK293 cells with no mtDNA from a dominant negative mitochondrial DNA polymerase construct and 143B rho0 and rho+ chronically depleted of mtDNA due to ethidium bromide. Only 143B were analyzed. The platform used was Illumina's Infinium 27k Human Methylation Beadchip. (PMID: 29668680, 30737248)

GSE45727: H9 hESCs with or without NLRP7 knockdown, both in undifferentiated and BMP4-induced states. The platform used was Illumina's Infinium 27k Human Methylation Beadchip. (PMID: 24105472)

GSE142439: human fibroblasts and endothelial cells with transient expression of OCT4, SOX2, KLF4, c-MYC, LIN28 and NANOG. The platform used was Illumina's Infinium MethylationEPIC. (PMID: 32210226)

GSE54848: reprogramming time course from human fibroblasts to iPSCs with OSKM. The platform used was Illumina's Infinium 27k Human Methylation Beadchip. (PMID: 25097266)

CANCER

GSE32393: breast samples from women with or without breast cancer. Cancer samples were used for the cancer comparison whereas healthy samples were used earlier for train/validation/test. The platform used was Illumina's Infinium 27k Human Methylation Beadchip. (PMID: 22346766)

GSE37988: liver samples from tumors or adjacent to tumors (two samples per patient). Cancer samples were used for the cancer comparison whereas healthy samples were used earlier for train/validation/test. The platform used was Illumina's Infinium 27k Human Methylation Beadchip. (PMID: 22976466)

GSE26126: prostate samples from normal and cancer tissue, and cultured prostate cells overexpressing DNMTs and EZH2. Cancer samples were used for the cancer comparison whereas healthy samples were used earlier for train/validation/test. The platform used was Illumina's Infinium 27k Human Methylation Beadchip. (PMID: 21521786)

GSE63384: lung samples from tumors or adjacent to tumors (two samples per patient). Cancer samples were used for the cancer comparison whereas healthy samples were used earlier for train/validation/test. The platform used was Illumina's Infinium 27k Human Methylation Beadchip. (PMID: 26134223)

GSE59157: kidney samples from tumor patients and healthy subjects. Cancer samples were used for the cancer comparison whereas healthy samples were used earlier for train/validation/test. The platform used was Illumina's Infinium 450k Human Methylation Beadchip. (PMID: 25134821)

E-GEOD-32867: lung tumor and adjacent non-tumor lung samples. Cancer samples were used for the cancer comparison whereas healthy samples were used earlier for train/validation/test. The platform used was Illumina's Infinium 27k Human Methylation Beadchip. (PMID: 22613842)

E-GEOD-30759: uterine cervix and cervical cancer samples. Cancer samples were used for the cancer comparison whereas healthy samples were used earlier for train/validation/test. The platform used was Illumina's Infinium 27k Human Methylation Beadchip. (PMID: 22346766, 22453031, 27103033)

E-GEOD-31979: breast tumor and adjacent non-tumor breast samples. Cancer samples were used for the cancer comparison whereas healthy samples were used earlier for train/validation/test. The platform used was Illumina's Infinium 27k Human Methylation Beadchip. (PMID: 21825015)

E-GEOD-77955: liver, colon, and small intestine samples from patients with and without cancer. Cancer samples were used for the cancer comparison whereas healthy samples were used earlier for train/validation/test. The platform used was Illumina's Infinium 450k Human Methylation Beadchip. (PMID: 27270421)

E-GEOD-52068: nasopharyngeal epithelial tissue from tumors or healthy controls. Cancer samples were used for the cancer comparison whereas healthy samples were used earlier for train/validation/test. The platform used was Illumina's Infinium 450k Human Methylation Beadchip. (PMID: 26443805, 28146149)

E-GEOD-49149: pancreatic ductal adenocarcinoma samples and normal adjacent tissues. Cancer samples were used for the cancer comparison whereas healthy samples were used earlier for train/validation/test.

The platform used was Illumina's Infinium 450k Human Methylation Beadchip. (PMID: 24500968, 26909576)

E-GEOD-39004: breast tissue samples with or without tumors. Cancer samples were used for the cancer comparison whereas healthy samples were used earlier for train/validation/test. The platform used was Illumina's Infinium 450k Human Methylation Beadchip. (PMID: 24316975, 30501643, 25016594)

GSE53051: samples from normal tissue and lung, pancreas, colon, thyroid, and breast cancer. Samples were used for the cancer comparison. The platform used was Illumina's Infinium 450k Human Methylation Beadchip. (PMID: 25191524)
