# Supplementary material for: A pan-tissue DNA-methylation epigenetic clock based on deep learning
Source: NPJ Aging. 2022 Apr 19;8(1):4. doi: 10.1038/s41514-022-00085-y (PMC9158789; doi:10.1038/s41514-022-00085-y)
Supplement: Supplementary file 4 — Importance values [file 41514_2022_85_MOESM4_ESM.pdf]

SupplementaryFile2_revised

	deep_pink_importance	deep_pink_importance	deep_pink_importance	deep_pink_importance	shap_importance	shap_importance	shap_importance	shap_importance
	median	mean	std	len	median	mean	std	len
**state_modes**								
**1.0**	3.5065444364502E-15	0.0016862766486502400	0.038308641559258900	9875	2.89386154580517E-07	0.004100140548513980	0.010318695500236500	9875
**2.0**	3.70369221968344E-15	0.004012199854296670	0.08058562598694220	1667	9.3800327688673E-07	0.005426885955420760	0.012228803693853000	1667
**3.0**	3.4376629207338E-15	0.003208798403865520	0.03001565071794000	1242	9.22604635434966E-07	0.008375896643943050	0.018296721293475000	1242
**4.0**	2.47012453951515E-15	0.014532680453407300	0.24960114250072700	517	6.79079513552913E-07	0.007957312826920600	0.021400902100254100	517
**5.0**	3.50255466830605E-15	0.0005814011139496840	0.006958874592984820	156	1.37423954266923E-07	0.003077444624068280	0.008390333320037140	156
**6.0**	2.960868440797E-15	0.0010548753358118600	0.01930653573295280	939	1.1173986928619E-07	0.0035517658531900100	0.01090919102598000	939
**7.0**	1.99788169668087E-15	0.0034063030945771900	0.019567721511338600	35	4.61421091327166E-08	0.0034927299154610700	0.010438484021500100	35
**8.0**	2.01634771074698E-16	2.67524217434576E-13	7.83634489618559E-13	11	9.26004099482457E-08	0.006447126090439150	0.011188357512928400	11
**9.0**	2.19288506050718E-15	0.006783028573025770	0.06087152007246600	120	3.07380394936733E-07	0.004248179128912560	0.011761971998472600	120
**10.0**	2.46292634619127E-15	0.003816839390798710	0.032587542784241900	296	1.42458610871271E-07	0.0029713681987225300	0.009466696042040920	296
**11.0**	2.49085522231763E-15	0.002153816055867650	0.016815068316845500	320	2.23313785173677E-07	0.007723610595825890	0.019912237361682700	320
**12.0**	2.35059795468258E-14	0.007998454470927110	0.03900030157104710	49	0.0016790752602669600	0.0146120718938227	0.03198188759680640	49
**13.0**	2.47118614058762E-14	1.39027209388933E-12	3.15960807278857E-12	29	1.01009739183996E-06	0.004720854791881710	0.010991379143837900	29
**14.0**	4.57059023486112E-15	0.004260424939373100	0.04045102955350080	1390	7.57377186500136E-07	0.007430287651607570	0.016330497384917600	1390
**15.0**	5.70328107752386E-15	0.01046260212757230	0.09161794539709640	278	8.46235268516986E-07	0.008721054031362890	0.021427906671223000	278
**16.0**	7.31441089217093E-15	0.007321801868513640	0.06991368707713090	926	8.41866544267151E-07	0.007592660946796800	0.018260952926027400	926
**17.0**	4.38679477565722E-15	0.0032440594004696900	0.047216031247879600	995	8.761872211062E-08	0.004357338959152660	0.01234476108668150	995
**18.0**	2.94221246178056E-15	0.00133535716171223	0.01801923965168090	2520	8.53497774033219E-08	0.003266349254635280	0.00998204326898277	2520
