# Supplementary material for: A pan-tissue DNA-methylation epigenetic clock based on deep learning
Source: NPJ Aging. 2022 Apr 19;8(1):4. doi: 10.1038/s41514-022-00085-y (PMC9158789; doi:10.1038/s41514-022-00085-y)
Supplement: Supplementary file 5 — Results details [file 41514_2022_85_MOESM5_ESM.pdf]

dataset	Tissue type	Data	n	Male proportion	Median age	Std age	MAE (Horvath)	MSE (Horvath)	R (Horvath)	Median error (Horvath)	MAE (AltumAge)	MSE (AltumAge)	R (AltumAge)	Median error (AltumAge)
E-GEOD-21232	pancreas	test	4	1.0	69.5	7.661	13.08	208.647	0.878	13.08	12.876	170.138	0.985	12.876
E-GEOD-27044	blood wbc	test	180	1.0	10.583	4.716	1.269	6.723	0.878	0.054	1.058	3.7	0.914	0.095
E-GEOD-30758	uterus	test	61	0.0	25.0	9.057	6.196	53.033	0.798	-5.388	3.583	23.277	0.872	-0.707
E-GEOD-30759	uterus	test	6	0.0	39.964	7.42	1.036	11.195	0.935	-0.913	6.242	42.681	0.743	-6.242
E-GEOD-30870	blood cord; blood whole	test	16	0.0	90.0	36.617	14.428	172.314	0.973	14.428	9.236	106.803	0.988	9.236
E-GEOD-31979	breast	test	6	0.0	50.0	2.687	10.657	165.011	0.039	-10.657	5.061	31.931	-0.703	-3.362
E-GEOD-32146	colon	test	4	0.5	14.5	5.624	2.153	39.851	0.149	0.088	2.954	22.569	0.607	2.065
E-GEOD-32149	blood whole	test	8	0.375	11.0	12.023	1.338	3.163	0.99	0.838	1.119	1.347	0.996	-0.415
E-GEOD-32396	blood wbc	test	12	0.0	54.5	6.764	5.153	95.113	0.487	4.142	2.21	55.58	0.324	-1.245
E-GEOD-32867	lung	test	24	0.125	69.0	10.458	3.8	17.362	0.924	-1.695	3.252	28.142	0.937	3.252
E-GEOD-34639	blood cd4+ t cell	test	20	0.9	0.0	0.497	0.132	0.122	0.876	-0.111	0.184	0.065	0.921	0.139
E-GEOD-36054	blood leukocyte	test	54	0.593	3.125	4.606	0.64	3.215	0.928	0.244	0.729	1.813	0.971	0.671
E-GEOD-36194	brain cerebellum; brain frontal cortex	test	289	0.433	48.0	27.911	5.675	133.641	0.915	-1.29	3.286	83.059	0.946	-0.557
E-GEOD-39004	breast	test	4	0.0	44.0	5.895	11.126	256.853	0.573	-11.126	4.241	212.246	0.091	-4.241
E-GEOD-40279	blood whole	test	263	0.483	65.0	14.127	3.823	35.396	0.921	1.481	3.725	34.948	0.924	2.213
E-GEOD-41169	blood whole	test	14	0.571	27.0	7.007	1.49	17.904	0.915	-0.822	1.258	4.242	0.957	-0.268
E-GEOD-41826	brain frontal cortex	test	31	0.484	35.0	15.946	2.355	24.96	0.967	-1.194	2.004	10.944	0.979	-0.312
E-GEOD-42700	buccal	test	22	0.5	0.0	0.737	0.779	1.376	0.745	-0.779	0.241	0.378	0.667	0.08
E-GEOD-43256	placenta	test	4	0.5	-0.585	0.041	0.196	0.122	0.837	-0.196	0.086	0.016	0.844	-0.086
E-GEOD-44712	placenta	test	16	0.688	-0.148	0.071	0.305	0.151	0.336	-0.305	0.318	0.104	0.24	0.318
E-GEOD-44763	blood whole	test	9	0.0	58.0	9.333	3.198	24.16	0.876	-0.426	2.599	17.972	0.938	-2.599
E-GEOD-45461	bone marrow	test	9	0.0	-0.345	0.0	0.088	0.015		0.088	0.043	0.004		0.043
E-GEOD-47513	adipose	test	5	0.0	29.0	3.286	14.587	221.725	0.142	-14.587	3.538	19.985	0.235	-3.538
E-GEOD-48325	liver	test	8	0.0	52.0	11.851	4.853	40.963	0.98	4.853	3.767	31.203	0.956	3.767
E-GEOD-48988	colon	test	68	0.0	66.0	9.367	4.31	39.826	0.788	-3.114	2.558	17.385	0.906	-0.443
E-GEOD-49064	blood pbmc	test	4	1.0	58.0	9.083	3.611	13.715	0.94	1.399	1.459	4.714	0.979	-0.611
E-GEOD-49149	pancreas	test	12	0.667	70.0	9.157	7.103	135.514	0.562	7.103	2.829	39.593	0.756	-0.082
E-GEOD-49393	brain prefrontal cortex	test	10	0.7	61.0	8.638	6.509	47.974	0.75	6.194	8.05	67.084	0.47	3.914
E-GEOD-50498	muscle	test	20	1.0	23.0	26.884	14.78	302.025	0.933	-9.847	2.638	21.308	0.989	1.207
E-GEOD-50660	blood whole	test	177	0.695	57.0	6.715	2.643	18.399	0.8	-0.14	2.439	15.276	0.836	1.012
E-GEOD-50759	buccal	test	20	0.4	11.5	7.201	4.954	41.501	0.93	4.954	2.559	18.087	0.84	1.451
E-GEOD-51032	blood buffy coat	test	338	0.222	54.175	7.154	2.836	22.837	0.792	0.279	3.004	18.783	0.816	0.886
E-GEOD-51388	blood whole	test	24	0.625	31.5	10.661	8.292	99.261	0.901	-8.292	1.149	10.593	0.963	0.84
E-GEOD-51954	dermis; epidermis	test	15	0.4	34.0	25.267	11.595	154.817	0.942	-10.258	2.478	26.81	0.989	1.317
E-GEOD-52068	nasopharyngeal	test	10	0.6	43.5	8.879	6.913	70.906	0.867	-6.913	4.456	34.646	0.78	0.333
E-GEOD-52588	blood whole	test	24	0.125	34.0	18.156	7.583	87.438	0.957	-7.044	2.873	21.272	0.972	-0.306
E-GEOD-53128	blood whole	test	18	0.0	63.606	8.063	7.66	123.121	0.576	7.66	4.077	89.573	0.524	4.077
E-GEOD-53162	brain cerebellum; brain prefrontal cortex; brain temporal cortex	test	9	0.889	26.0	15.235	9.302	69.401	0.948	-9.302	5.503	30.25	0.934	-3.588
E-GEOD-53740	blood whole	test	78	0.333	69.5	11.106	8.24	133.27	0.778	8.24	3.678	48.531	0.829	2.232
E-GEOD-54211	buccal	test	9	0.0	55.0	4.522	5.396	71.613	0.254	4.793	2.527	69.527	0.264	-1.557
E-GEOD-54399	blood cord; placenta	test	18	0.667	0.0	0.0	0.207	0.113		-0.207	0.224	0.076		0.224
E-GEOD-54690	blood whole	test	8	1.0	43.5	6.518	4.186	28.562	0.903	-4.186	2.082	8.522	0.954	0.441
E-GEOD-55438	placenta	test	20	0.0	-0.397	0.247	0.084	0.048	0.811	0.004	0.166	0.043	0.589	0.035
E-GEOD-56342	bronchiole	test	10	0.5	63.5	5.426	11.322	134.61	0.695	11.322	4.502	29.885	0.332	0.896
E-GEOD-56515	adrenal; amnion; muscle; pancreas	test	14	0.0	-0.422	0.097	0.834	1.937	0.635	-0.834	0.203	0.471	0.598	-0.203
E-GEOD-56553	blood pbmc	test	15	0.333	26.1	8.104	4.513	28.953	0.96	4.513	1.5	2.76	0.989	0.791
E-GEOD-57484	blood whole	test	5	1.0	10.43	0.178	1.061	10.228	-0.651	-1.061	2.981	7.914	0.04	-2.981
E-GEOD-57767	placenta	test	14	0.0	0.0	0.0	0.178	0.179		-0.149	0.368	0.127		0.368
E-GEOD-58045	blood whole	test	69	0.0	59.904	7.756	3.412	24.473	0.826	1.983	1.961	15.566	0.863	-0.037
E-GEOD-58119	blood serum	test	113	0.0	63.0	6.326	13.986	313.737	0.112	13.535	6.202	60.705	-0.099	1.193
E-GEOD-59457	brain frontal; brain hippocampus; brain midbrain; brain occipital; brain temporal	test	13	0.538	53.0	10.393	9.21	112.635	0.897	9.21	1.403	9.568	0.971	-0.324
E-GEOD-59509	blood whole; saliva; vaginal swab	test	11	0.727	38.0	13.514	3.703	17.94	0.961	2.301	1.137	10.069	0.974	-0.195
E-GEOD-59592	blood whole	test	48	0.562	0.375	0.0	0.617	0.937		-0.617	0.448	0.223		0.448
E-GEOD-61107	brain frontal cortex	test	10	0.8	75.0	9.47	15.009	272.862	0.85	15.009	5.837	60.882	0.878	5.837
E-GEOD-61257	adipose	test	13	0.385	45.0	12.749	6.313	52.137	0.962	-6.313	1.906	12.735	0.964	0.991
E-GEOD-61258	liver	test	32	0.5	54.0	16.165	6.3	89.32	0.872	-0.466	2.992	36.508	0.936	0.545
E-GEOD-61259	muscle	test	11	0.364	48.0	8.202	4.06	23.625	0.808	0.256	4.512	36.155	0.844	4.13
E-GEOD-61380	brain frontal cortex	test	6	0.667	36.5	11.776	4.541	42.262	0.841	-2.183	3.66	23.521	0.925	-1.087
E-GEOD-61454	adipose; liver; muscle	test	108	0.231	42.0	9.581	6.386	66.428	0.764	-5.363	2.964	23.722	0.865	-1.014
E-GEOD-62219	blood leukocyte	test	24	0.0	2.0	1.767	1.589	8.059	0.751	-1.236	0.994	2.411	0.698	0.58
E-GEOD-62867	blood vessel; blood whole	test	8	1.0	53.5	4.635	5.535	48.198	0.796	-3.629	1.942	10.826	0.811	-0.057
E-GEOD-63106	cartilage	test	24	0.417	68.45	8.411	3.603	40.362	0.667	-1.264	3.994	32.735	0.755	-0.822
E-GEOD-63347	brain cerebellum; brain frontal cortex; brain hippocampus; brain midbrain; brain occipital cortex; brain temporal cortex	test	23	0.348	59.0	8.722	10.359	135.815	0.83	10.359	1.235	7.228	0.958	0.837
E-GEOD-64495	blood whole	test	43	0.256	45.1	15.477	2.718	14.73	0.971	1.223	2.223	9.115	0.981	-0.182
E-GEOD-64511	adrenal; bone; bone marrow; brain caudate nucleus; brain cerebellum; brain cingulate gyrus; brain frontal cortex; brain l	test	149	0.349	80.0	25.566	16.078	445.514	0.887	15.893	3.165	54.128	0.963	1.615
E-GEOD-64940	blood cord	test	87	0.54	0.0	0.0	0.121	0.045		0.046	0.249	0.085		0.245
E-GEOD-65638	blood whole	test	7	0.0	25.0	4.435	3.724	108.105	-0.088	-3.459	2.472	52.196	0.079	-0.354
E-GEOD-67024	adipose	test	6	0.0	40.0	12.24	17.474	423.124	0.126	-17.474	10.662	139.09	0.306	-0.311
E-GEOD-67444	blood whole	test	28	0.607	1.0	1.693	1.571	19.836	0.716	-1.571	0.686	6.261	0.706	0.246
E-GEOD-67705	blood whole	test	18	1.0	52.5	11.093	8.773	130.823	0.91	-8.773	2.522	8.922	0.968	-0.409
E-GEOD-71245	blood b cells; blood cd4 t cells; blood cd8 t cells; blood monocyte; blood pbmc	test	10	0.0	49.0	8.149	4.395	90.829	0.304	-2.821	4.811	20.616	0.882	1.637
E-GEOD-71678	placenta	test	132	0.5	0.0	0.0	2.009	74.601		-2.009	0.319	0.106		0.319
E-GEOD-71955	blood cd4 t cells; blood cd8 t cells	test	25	0.04	50.0	9.453	6.14	126.576	0.452	-2.202	3.368	73.278	0.561	0.682
E-GEOD-72338	blood monocyte; blood neutrophil	test	8	0.5	23.0	10.283	1.779	6.349	0.977	-0.46	1.693	4.997	0.987	-1.2
E-GEOD-72556	saliva	test	38	0.447	3.5	0.792	0.517	1.664	0.082	0.413	0.864	1.903	0.207	0.629
E-GEOD-73103	blood whole	test	142	0.556	20.0	4.625	4.114	38.791	0.709	-3.93	1.571	7.421	0.809	-0.291
E-GEOD-73377	placenta	test	15	0.0	0.0	0.0	0.198	0.273		0.037	0.372	0.246		0.297
E-GEOD-73832	intestine	test	12	0.417	61.0	14.683	8.648	177.51	0.743	7.451	7.045	69.895	0.869	-5.169
E-GEOD-74738	placenta	test	12	0.75	-0.024	0.023	0.112	0.029	0.412	0.041	0.419	0.202	0.598	0.419
E-GEOD-75248	placenta	test	130	0.485	0.0	0.0	3.121	10.937		-3.121	0.322	0.105		0.322
E-GEOD-76105	brain superior temporal gyrus	test	14	0.5	78.5	7.832	6.926	62.107	0.799	-6.926	4.141	30.829	0.788	-2.089
E-GEOD-77445	blood whole	test	34	0.588	25.0	15.721	7.702	85.956	0.819	-3.98	4.969	77.989	0.833	-0.859
E-GEOD-77955	colon; liver	test	6	0.833	60.0	11.475	5.546	266.227	0.05	5.452	5.371	139.131	0.355	0.805
E-GEOD-79056	blood cord	test	15	0.4	-0.121	0.096	0.474	0.214	0.686	-0.474	0.091	0.013	0.611	0.086
E-GEOD-83334	blood cord; blood whole	test	12	0.5	5.0	2.357	0.829	1.931	0.88	0.083	0.785	1.339	0.923	0.273
E-MTAB-2344	blood wbc	test	3	0.667	77.0	8.014	5.534	69.766	0.699	-5.277	6.519	35.167	0.706	0.784
E-MTAB-2372	blood wbc	test	19	0.421	49.0	9.831	16.349	302.658	0.869	-16.349	2.497	52.962	0.696	0.043
E-MTAB-487	cd14+ monocytes; cd16+ granulocytes; cd34+ hematopoietic progenitor cells; fibroblasts ; keratinocytes ; mesenchym	test	13	0.0	21.0	13.305	3.869	63.363	0.913	-3.869	3.019	54.677	0.936	-3.019
E-MTAB-625	epidermis	test	12	0.0	45.5	10.332	5.426	66.171	0.749	-4.725	5.496	63.922	0.666	2.781
GSE108213	breast	test	34	0.0	56.5	7.614	11.032	130.355	0.814	-11.032	2.459	13.729	0.883	0.382
GSE115797	skin	test	10	0.7	31.5	9.837	7.94	150.114	0.595	7.713	6.643	70.292	0.699	-3.383
GSE148000	sputum	test	3	0.667	39.0	9.568	5.936	56.361	0.962	-5.936	3.82	16.194	0.95	3.82
GSE15745	brain pons; brain temporal cortex	test	101	0.604	45.0	26.176	3.171	50.708	0.963	-0.663	2.569	47.179	0.965	-0.059
GSE17448	bone marrow	test	7	0.0	25.0	22.173	9.69	100.321	0.915	-9.28	6.862	70.842	0.955	-6.862
GSE19711	blood whole	test	110	0.0	65.0	7.017	4.104	34.882	0.729	2.279	3.103	24.048	0.723	0.697
GSE20236	blood whole	test	38	0.0	63.0	5.295	7.566	75.104	0.667	7.566	3.336	19.249	0.783	3.054
GSE20242	blood cd14+ monocyte; blood cd4+ t cell	test	16	0.312	31.0	13.123	3.06	41.175	0.889	0.669	1.909	24.56	0.954	-1.105
GSE22595	dermis	test	6	0.0	12.5	23.576	10.849	88.372	0.952	-6.406	6.084	74.567	0.974	-6.007
GSE25892	buccal	test	44	0.341	15.0	0.0	1.68	7.717		0.842	0.83	1.988		-0.772
GSE26126	prostate	test	62	1.0	61.5	7.195	5.261	62.832	0.42	0.376	6.121	64.267	0.317	0.607
GSE27317	blood cord	test	68	0.544	0.0	0.0	0.123	0.057		0.059	0.231	0.109		0.216
GSE28746	saliva	test	34	1.0	37.0	8.468	2.437	11.72	0.929	0.501	2.161	8.385	0.942	-0.892
GSE32393	breast	test	10	0.0	50.745	12.669	7.434	88.165	0.697	-0.908	6.061	91.757	0.668	-0.593
GSE34035	saliva	test	79	0.684	28.0	9.064	3.516	38.893	0.807	1.786	2.636	21.472	0.865	-0.719
GSE34257	blood cord	test	34	0.559	0.0	0.369	0.163	0.217	0.528	-0.072	0.37	0.28	0.482	0.36
GSE36166	muscle	test	18	1.0	25.0	0.0	16.457	287.679		-16.457	1.23	54.021		-0.228
GSE36642	blood cord; placenta	test	50	0.48	-0.077	0.033	0.359	21.297	-0.124	-0.359	0.202	0.133	-0.143	0.06
GSE36812	blood cord	test	20	0.0	-0.011	0.018	0.465	112.837	0.088	-0.465	0.17	114.057	0.092	-0.036
GSE37008	blood pbmc	test	38	0.263	33.0	5.144	3.417	18.141	0.745	1.272	3.026	14.951	0.676	-0.232

**GSE37988**	liver	test	23	0.87	56.0	14.037	4.528	39.334	0.902	-0.756	5.441	46.409	0.885	0.361
**GSE38291**	muscle	test	5	0.4	65.0	6.524	16.209	325.808	-0.442	16.209	2.798	16.64	0.783	0.644
**GSE38608**	brain cerebellum; brain occipital cortex	test	7	1.0	8.0	10.335	2.421	28.722	0.982	-2.421	1.003	13.796	0.993	-1.003
**GSE38873**	cerebellum	test	21	0.0	50.0	7.737	3.732	21.175	0.833	0.992	4.336	27.48	0.741	-1.196
**GSE40360**	brain frontal lobe	test	8	0.5	71.5	7.952	18.152	335.667	0.731	18.152	4.875	63.177	0.784	4.875
**GSE41037**	blood whole	test	158	0.538	37.0	17.748	2.624	25.566	0.961	0.769	2.909	19.621	0.969	-0.952
**GSE42861**	blood leukocyte	test	134	0.313	54.0	10.979	3.572	26.349	0.898	-1.68	3.046	19.814	0.916	0.562
**GSE49904**	blood buffy coat	test	29	0.276	57.0	14.61	4.659	24.742	0.944	-1.365	3.661	24.276	0.944	-1.679
**GSE49905**	brain cerebral cortex	test	32	0.656	18.5	14.103	3.242	30.721	0.932	-0.24	3.085	57.223	0.853	-1.562
**GSE49907**	kidney	test	34	0.676	58.0	10.387	5.548	53.151	0.747	-2.486	3.583	17.6	0.917	-1.258
**GSE49908**	muscle	test	21	1.0	50.0	16.656	9.052	114.112	0.835	-0.261	4.208	49.371	0.931	-1.406
**GSE56606**	blood cd14+ monocyte	test	9	0.222	35.0	9.754	2.196	24.022	0.922	-1.67	1.849	16.8	0.951	-1.488
**GSE57285**	blood whole	test	17	0.0	46.0	13.754	7.456	375.678	0.273	5.295	8.85	354.881	0.246	3.522
**GSE59157**	kidney	test	14	0.357	35.0	17.722	24.46	873.669	0.854	24.46	11.201	236.837	0.647	6.943
**GSE59274**	placenta	test	20	0.55	-0.042	0.07	0.327	0.872	-0.384	0.304	0.324	0.927	-0.426	0.313
**GSE63384**	lung	test	14	0.571	63.5	10.727	5.378	34.266	0.86	-2.781	1.936	25.782	0.884	-0.022
**GSE69176**	blood cord	test	61	0.541	0.0	0.0	0.228	29.466		-0.22	0.237	24.487		0.222
**GSE77241**	brain; brain frontal lobe	test	2	1.0	16.5	16.5	0.852	1.044	1.0	0.852	2.686	8.148	1.0	0.966
**GSE90124**	skin	test	129	0.0	60.61	9.359	4.961	49.059	0.793	3.322	2.701	24.641	0.853	0.3
**GSE92767**	saliva	test	22	1.0	49.0	14.116	3.813	20.559	0.963	0.681	2.194	13.752	0.982	1.659
**GSE99624**	blood whole	test	6	0.5	60.5	8.641	2.915	12.734	0.946	-1.955	2.622	12.82	0.945	1.234
**TCGA_BRCA**	breast	test	46	0.0	58.742	15.906	7.931	87.95	0.901	-7.12	4.35	73.691	0.858	0.776
**TCGA_COAD**	colon	test	30	0.633	68.084	14.226	5.389	59.121	0.888	2.898	4.846	50.704	0.906	-0.654
**TCGA_HNSC**	head and neck	test	20	0.85	62.329	11.801	6.507	66.611	0.825	-3.911	3.784	22.139	0.936	-0.73
**TCGA_KIRC**	kidney	test	143	0.657	63.113	11.026	3.624	46.403	0.812	1.637	3.174	34.177	0.857	0.782
**TCGA_KIRP**	kidney	test	20	0.7	63.837	12.938	5.138	55.887	0.884	4.376	3.322	26.077	0.945	2.809
**TCGA_LIHC**	liver	test	19	0.632	65.834	19.426	5.724	140.448	0.871	5.673	3.016	35.235	0.958	-0.107
**TCGA_LUAD**	lung	test	22	0.455	67.173	10.241	5.57	45.686	0.828	-4.063	3.007	18.206	0.909	-1.621
**TCGA_LUSC**	lung	test	28	0.75	71.155	7.708	3.953	26.476	0.747	0.116	2.514	21.976	0.835	1.492
**TCGA_PRAD**	prostate	test	20	1.0	65.962	5.833	3.175	26.514	0.642	2.476	3.247	20.644	0.65	1.231
**TCGA_STAD**	stomach	test	11	0.545	72.723	9.9	4.011	35.755	0.827	-0.623	4.444	29.763	0.84	2.15
**TCGA_THCA**	thyroid	test	23	0.261	47.357	17.372	10.166	121.747	0.942	-10.166	4.719	46.629	0.934	-1.982
**TCGA_UCEC**	uterus	test	13	0.0	62.163	9.84	12.347	295.142	-0.316	-5.249	3.977	81.868	0.449	-0.904
